# Molecular and Evolutionary Analysis of NEAr-Iron Transporter (NEAT) Domains

**DOI:** 10.1371/journal.pone.0104794

**Published:** 2014-08-25

**Authors:** Erin S. Honsa, Anthony W. Maresso, Sarah K. Highlander

**Affiliations:** 1 Department of Molecular Virology and Microbiology, Baylor College of Medicine, Houston, Texas, United States of America; 2 Genomic Medicine, The J. Craig Venter Institute, La Jolla, California, United States of America; Institut Pasteur Paris, France

## Abstract

Iron is essential for bacterial survival, being required for numerous biological processes. NEAr-iron Transporter (NEAT) domains have been studied in pathogenic Gram-positive bacteria to understand how their proteins obtain heme as an iron source during infection. While a 2002 study initially discovered and annotated the NEAT domain encoded by the genomes of several Gram-positive bacteria, there remains a scarcity of information regarding the conservation and distribution of NEAT domains throughout the bacterial kingdom, and whether these domains are restricted to pathogenic bacteria. This study aims to expand upon initial bioinformatics analysis of predicted NEAT domains, by exploring their evolution and conserved function. This information was used to identify new candidate domains in both pathogenic and nonpathogenic organisms. We also searched metagenomic datasets, specifically sequence from the Human Microbiome Project. Here, we report a comprehensive phylogenetic analysis of 343 NEAT domains, encoded by Gram-positive bacteria, mostly within the phylum Firmicutes, with the exception of *Eggerthella* sp. (Actinobacteria) and an unclassified Mollicutes bacterium (Tenericutes). No new NEAT sequences were identified in the HMP dataset. We detected specific groups of NEAT domains based on phylogeny of protein sequences, including a cluster of novel clostridial NEAT domains. We also identified environmental and soil organisms that encode putative NEAT proteins. Biochemical analysis of heme binding by a NEAT domain from a protein encoded by the soil-dwelling organism *Paenibacillus polymyxa* demonstrated that the domain is homologous in function to NEAT domains encoded by pathogenic bacteria. Together, this study provides the first global bioinformatics analysis and phylogenetic evidence that NEAT domains have a strong conservation of function, despite group-specific differences at the amino acid level. These findings will provide information useful for future projects concerning the structure and function of NEAT domains, particularly in pathogens where they have yet to be studied.

## Introduction

All bacteria must acquire iron from their environment to survive, with mammalian pathogens exploiting host iron reservoirs during an infection [1]. However, to protect against the toxic effects of free iron, and as a form of “nutritional immunity”, the host sequesters approximately 80% of iron within heme, which is further coordinated by proteins such as hemoglobin [2], [3]. It should be noted that heme is primarily used by mammals as a cofactor in several proteins, including hemoglobin (oxygen transport), myoglobin (oxygen storage), and peroxidases (*e.g.*, glutathione peroxidase, which protects mammalian cells against oxidative stress [4]). In order to obtain iron from the host, bacteria exploit hemoglobin by targeting heme stored within. The mechanism of heme-iron acquisition in Gram-negative pathogens has been characterized in detail: secreted bacterial proteins bind free heme spontaneously released from hemoglobin and then they interact with bacterial TonB-dependent cell surface receptors, where heme is imported into the periplasm [5], [6], [7], [8], [9]. Heme is then passed through the inner membrane by an ABC-transporter complex [10], [11], [12], [13], [14]. However, due to differences in cell envelope architecture, the action of heme-iron capture and import by Gram-positive organisms is most likely mediated by an alternative mechanism.

In 2002, a bioinformatics study examined genes mapping near putative Fe^3+^ transporters in the genomes of Gram-positive bacteria [15]. Here, the first description of the NEAr-iron Transporter (NEAT) domain was reported. NEAT is domain with a predicted β-strand secondary structure, and NEAT-containing proteins were proposed to be cell membrane anchored and exposed to the surface of the cell. NEAT domains were originally identified as being encoded by the genomes of several Gram-positive pathogens (*e.g.*, *Bacillus anthracis*, *Staphylococcus aureus*, *Streptococcus pyogenes*, *Clostridium perfringens* and *Listeria monocytogenes*) and two non-pathogens (*Bacillus halodurans* and *Listeria innocua*). This *in silico* study reported the initial identification of NEAT domains and provided information critical to perform later biochemical, structural and biophysical characterization of domain function. Indeed, studies performed over the past ten years have shown that NEAT domains enable Gram-positive bacterial proteins to acquire heme-iron from host hemoglobin or the haptoglobin-hemoglobin complex, contrary to the initial proposed role of NEAT domains as a siderophore-binding [15], [16], [17], [18]. NEAT proteins are covalently anchored to the cell wall (not to the cell membrane as previously proposed [15]) in *B. cereus*, *L. monocytogenes*, *S. aureus*, and *S. pyogenes*, and they are secreted and cell wall-anchored in *B. anthracis* [17], [19], [20], [21], [22], [23], [24], [25], [26], [27], [28], [29], [30]. The NEAT proteins expressed by these pathogens function together to scavenge heme from host hemoproteins such as hemoglobin and transfer it to and through the cell surface for delivery into the bacterial cytosol where iron is released. For comprehensive reviews on the *S. aureus* and *B. anthracis* NEAT systems, see Grigg *et al.* 2010 and Honsa and Maresso 2011, respectively.

NEAT domains are conserved heme binding modules, thought to be exclusive to Gram-positive bacteria, although the restriction based on this classification has not been confirmed and has not been addressed since the initial 2002 study [15]. While the primary amino acid sequence of the domains can vary, all NEAT domains are highly similar at a secondary structure level. NEAT domains must be composed of eight β-strands and a small 3_10_-helix (also known as the lip-region or alpha-helix) that fold to form a hydrophobic heme-binding pocket [31]. The biochemical functions and roles of NEAT proteins in disease and virulence have been studied in only five Gram-positive pathogens: *B. anthracis*, *B. cereus*, *L. monocytogenes*, *S. aureus* and *S. pyogenes*. For example, when certain genes encoding NEAT proteins are deleted in *S. aureus* (*isdC*), *B. anthracis* (*hal*) or *B. cereus* (*ilsA*), the bacteria cannot survive when hemoglobin is the sole iron source, and some of these mutant strains exhibit decreased virulence in murine or insect models of infection [21], [24], [28], [32], [33]. Recent studies have also exploited NEAT domains as potential vaccine candidates, due to their potent immunogenic properties [34], [35]. However, the breadth of NEAT heme-acquisition systems employed by bacterial pathogens remains unknown, as no data are currently available to confirm whether NEAT proteins are exclusive to these five Gram-positive pathogens. Furthermore, there is a lack of information concerning the level of conservation of NEAT proteins and domains at the species level. Therefore, the major goal of this study was to identify new NEAT domains by sequence mining, followed by phylogenetic clustering to investigate their overall conservation and distribution within the bacterial kingdom. We successfully identified 343 putative NEAT domains encoded by 82 Gram-positive bacterial species, most of which are new NEAT members. Mining of human metagenomic sequences failed to reveal novel NEAT sequences. In addition, we report that the heme binding function of NEAT domains is conserved, as we demonstrate that an environmental bacterium encodes a NEAT domain that can bind heme. We present homology-based modeling visualization of the first predicted NEAT domain from an anaerobic pathogen, *Clostridium botulinum*. In particular, we were interested in identifying new NEAT domains encoded by additional pathogenic species, to provide information needed for future studies that focus on iron acquisition systems in clinically relevant bacteria.

## Results and Discussion

### Identification of putative NEAT domains

**Figure 1:**
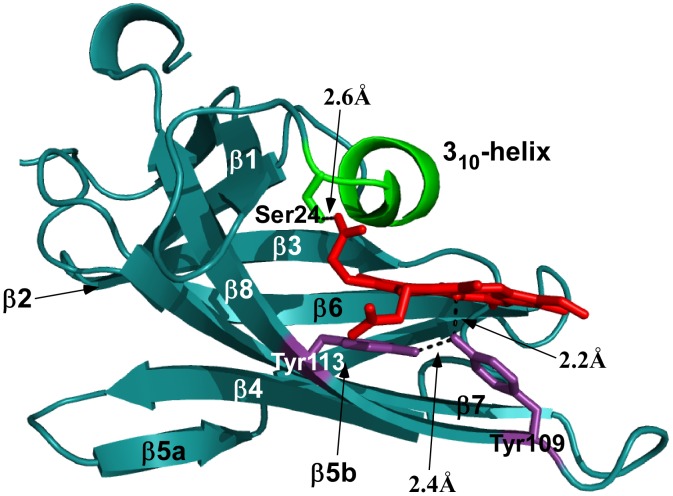
Structure of the *B. anthracis* IsdX1 NEAT domain. The ribbon structure of the heme-bound form of IsdX1 is shown. NEAT domains share a conserved β-barrel fold including a 3_10_-helix (green) that conventionally begins with a serine and ends with a tyrosine, and eight β-strands (teal). The two tyrosine residues (Tyr-109 and Tyr-113) within the heme-binding sequence YXXXY are indicated in purple. Tyr-109 non-covalently binds to the iron atom within the heme molecule at a predicted distance of 2.2 Å. Tyr-113 H-bonds with Tyr-109 as indicated by the dotted black line (2.4 Å). Ser-24 (green) H-bonds with the buried propionate group of the heme porphyrin, increasing binding affinity (2.6 Å). PDB code: 3SIK; [36].

To illustrate the features of the conserved NEAT domain, a structure of the *B. anthracis* IsdX1 NEAT protein is shown in Figure 1 (PDB code: 3SIK; [36]). NEAT domains can bind heme and/or hemoglobin, extract heme from hemoglobin by a physical interaction, and undergo NEAT-NEAT heme transfer events [17], [37], [38], [39], [40]. These functions are based on conserved, specific secondary structural regions of the NEAT domain, as well as critical amino acids within the heme-binding pocket. The 3_10_-helix (canonically SXXXXY), is a completely conserved sub-structure of all NEAT domains that lies on top of the distal side of heme. This functional region is necessary for heme binding by the *B. anthracis* IsdX1 NEAT protein, and is essential for heme extraction from hemoglobin by the *B. anthracis* IsdX2 NEAT protein [36], [40]. A glutamine at the fourth position within the 3_10_-helix is critical for IsdX2 to scavenge heme, and this activity is attributed to its amide side chain [40]. The second functional region, the “heme-binding signature”, is comprised of five amino acids within the heme-binding pocket on the eighth β-strand, and the motif generally begins and ends with a tyrosine (Figure 1). The presence of these two tyrosine residues correlates with the proposal that a NEAT domain should bind heme, as the first tyrosine non-covalently binds to the iron atom, and the second tyrosine hydrogen bonds (H-bonds) to the first tyrosine [17], [27], [41]. These interactions allow a strong coordination between the NEAT domain and heme. Additionally, some proteins contain more than one non-identical NEAT domain within the full-length sequence, such as the *S. aureus* IsdB (2 domains) and IsdH (3 domains) proteins, the *B. anthracis* IsdX2 protein (5 domains), and the *S. pyogenes* Shr protein (2 domains) [17], [22], [23], [25], [27], [33], [41], [42], [43], [44]. Both the 3_10_-helix sequence (SXXXXY) and the heme-binding sequence on the eighth β-strand (YXXXY) are important for NEAT function, at least in those NEAT domains that have been studied so far. Thus, they must be considered when analyzing other NEAT domains and when generating predictions about the role of NEAT proteins in heme-iron acquisition and their possible role in bacterial pathogenesis.

**Figure 2:**
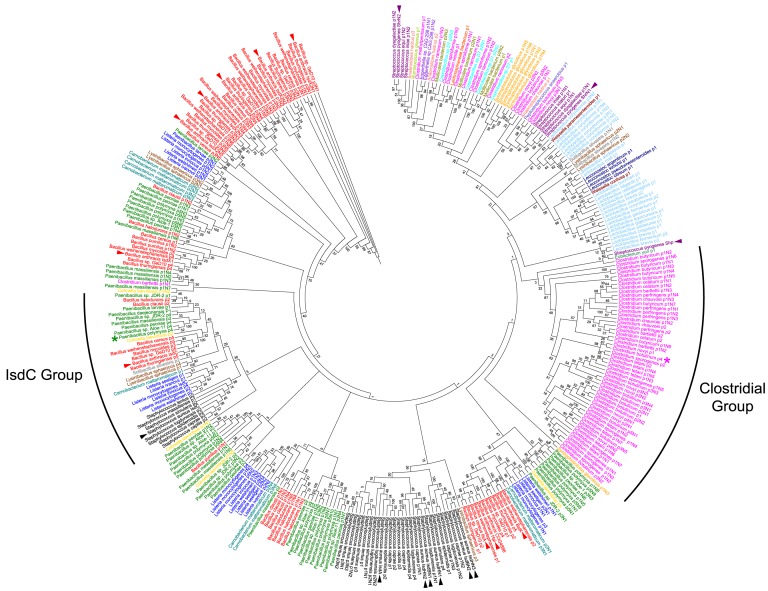
Phylogenetic tree demonstrating the relationship of the 343 identified putative NEAT domains. Each unique NEAT domain is included and each genus is indicated by a different color. Branch lengths are not representative phylogenetic distance; numbers on branches are bootstrap values obtained from 100 replicates. Colored arrowheads point to NEAT domains that have been previously characterized. The black arches indicate NEAT Groups discussed chosen for analysis. The green and magenta asterisks indicate Pp-p4 and Cb-p4, which are described in detail in the text.

Protein sequence mining allowed us to identify 343 putative NEAT domains within 185 proteins (Figure 2; Table S1). All of the sequences were identified from protein sequences in the National Center for Biotechnology Information (NCBI) Non-redundant protein database (nr). We also searched the annotated HMP metagenomic sequences (HMGI; www.hmpdacc.org) and found homologues only in samples corresponding to the vaginal sites (posterior fornix, mid vagina and vaginal introitus: total of 26 incomplete hits to a cell surface protein in *Lactobacillus crispatus*), and skin sites (anterior nares) one 100% homologue to IsdC; left retroauricular crease: one partial hit to an iron regulated cell surface protein in *Staphylococcus capitis*; right retroauricular crease: one partial hit to a cell surface protein in *Staphylococcus caprae*. Identical sequences were discovered in the search of the nr database so these did not contribute any new diversity to the NEAT family. All proteins were predicted to be secreted or cell-surface associated, with many possessing sortase motifs for covalent anchoring to the cell wall [45], [46], [47], [48]. No cytosolic proteins were identified, which is consistent with the recognized role of NEAT proteins in nutrient acquisition from the extracellular environment. Some proteins also possessed S-layer homology (SLH) domains that enable non-covalent anchoring of a protein to an S-layer, a crystalline structure that surrounds the capsule of various bacteria during certain growth conditions [49], [50], [51]. Once such example is the previously characterized *B. anthracis* BslK protein, which possesses a single heme-binding NEAT domain, and three SLH-domains (Figure 2, Table S1; [26]).

The 185 proteins are encoded by 82 bacterial species, all of which are members of the Firmicutes, with the exception of *Eggerthella* sp., which is a member of the Actinobacteria and the unclassified Mollicutes organism, which is a member of the Tenericutes (Table S2). These results suggest that NEAT domains are heme-acquisition modules that are almost exclusive to the Firmicutes phylum of Gram-positive bacteria. Many of the species identified in this study are characterized as environmental organisms (commonly found in soil or water), and have not been shown to be associated with mammalian hosts (Table S2). For example, *Paenibacillus polymyxa* is a nitrogen-fixing rhizobacterium that invades plant roots to aid in the growth of crops, and as a result is commonly used as an agricultural inoculant [52], [53], [54], [55]. Together, these data indicate that genes encoding NEAT proteins have remained present in the genomes of Gram-positive bacteria as they evolved to inhabit different ecological niches, whether in the environment or within mammalian hosts.

### Phylogenetic and functional NEAT groups

Phylogenetic mapping of the 343 NEAT domains to an unrooted tree (Figure 2) revealed some expected clustering, such as clusters with high bootstrap values within a species likely due to gene duplication [*e.g.* in *Lactobacillus coryniformis* (light blue text) or *Syntrophobotulus glycolicus* (orange text)]. Also, clusters of NEAT domains were identified that included members that have been the subject of previous functional studies such as: i) a staphylococcal cluster that contains the IsdA, IsdB and IsdH heme-acquisition domains from *S. aureus* (black text), which now also includes NEAT domains from coagulase negative staphylococci [56], [57]; ii) and a large cluster of *Bacillus* NEAT domains (red text) that resemble those in *B. anthracis* IsdX2, which contains five or six NEAT domains. The multi-NEAT IsdX2 protein is unique, as it is the only secreted NEAT protein and it possesses multiple domains that selectively acquire heme from hemoglobin (hemophores). Other members from the *Bacillus* genus cluster near the IsdX2 NEATs so are likely hemophore proteins. In addition, a cluster containing *B. anthracis* IsdX1 (single NEAT-containing protein) includes members of the Bacillales family, which are also likely hemophores. These two groups surround clusters of domains such as those in the *Paenibacillus* genus (Figure 2, green text) may also possess similar hemophore properties. Initial Pfam analysis did not detect secretion signals in any of the *Paenibacillus* NEAT proteins, so further biochemical analysis will be required to determine the actual localization of these proteins. The phylogenetic tree also revealed an IsdC Group, which contains the well-studied *B. anthracis* and *S. aureus* IsdC proteins, plus members from other genera. New clusters of uncharacterized NEAT domains were revealed, most significant of which is a large cluster of 51 NEAT domains from seven clostridial species (Figure 2, magenta text) plus a cluster of 50 NEAT domains that belong to the Lactobacillales order (light blue text). The IsdC and Clostridial Groups are discussed in more detail below.

#### The IsdC NEAT Group

This group contains predicted NEAT domains that are phylogenetically related to the IsdC from *S. aureus* and *B. anthracis* (Sa-IsdC and Ba-IsdC, Figure 2). The IsdC Group includes domains from 35 proteins encoded by 33 bacterial species within the class Bacilli. All proteins within this group contain a single IsdC NEAT domain. The IsdC Group is unique in our analysis, since there is a strong clustering of NEAT-proteins from diverse species and NEAT systems (*i.e.*, *B. anthracis*, *S. aureus*, *B. cereus*, *L. monocytogenes*), which is not demonstrated in any other region of the tree (Figure 2). Other clusters are genus- or family-specific, such as the IsdX2 Group of *Bacillus* species, the *Staphylococcus* Group and the Clostridial Group. Therefore, while different genera may have developed specialized NEAT proteins, many have retained the fundamental, critical IsdC-like protein.

IsdC proteins are cell wall-anchored via a sortase B motif, and can bind heme in a dose-dependent manner [31], [32], [38], [58], [59]. They also actively accept heme from other NEAT proteins of the same species [27], [59], [60], [61]. It is proposed that IsdC is essential for heme to traverse the cell wall, as Sa-IsdC has been shown to transfer heme to a cell membrane ABC-transporter complex. IsdC is the only known NEAT-protein capable of acquiring heme from upstream heme scavengers, or directly from the extracellular environment, traversing heme through the thick cell wall, and actively transferring it to a membrane complex. Therefore, it is proposed that IsdC is the central conduit of known NEAT heme acquisition system in bacterial pathogens [61]. For a bacterial species to employ NEAT-mediated heme acquisition, it must express an IsdC-like protein.

**Figure 3:**
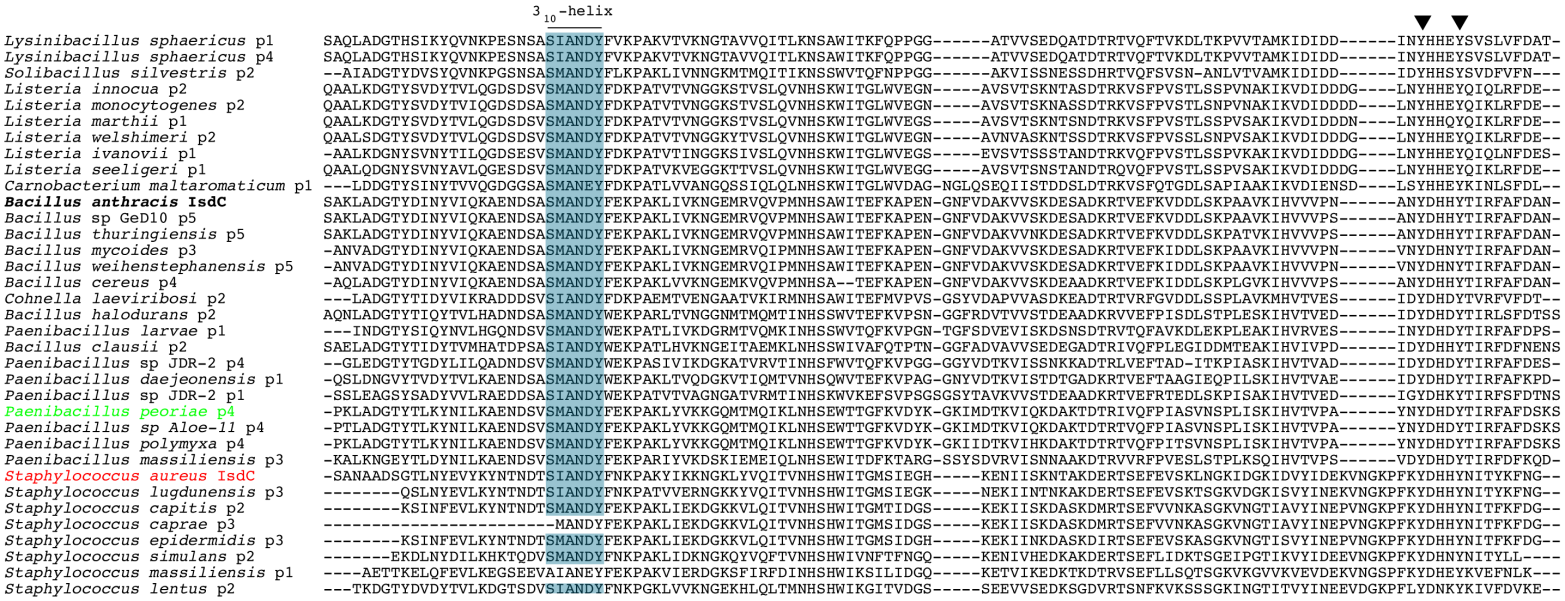
Alignment of IsdC Group NEAT domains. ClustalX generated alignment of the 35 IsdC Group NEAT domains. The 3_10_-helix is boxed in blue, and the arrowheads indicate the tyrosine residues within the heme-binding signature sequence. The two IsdC proteins of known function, from *B. anthracis* and *S. aureus*, are highlighted in black and red text, respectively. The putative IsdC-like NEAT from *P. polymyxa* is highlighted in green.

We therefore propose that IsdC-like NEAT proteins possess a conserved, fundamental role in NEAT-mediated heme acquisition. The NEAT domains within the IsdC Group are highly homologous and the heme-binding sequences of the 35 IsdC-like NEAT domains are nearly identical, with all possessing the two tyrosine residues required for heme binding (Figure 3). Since the newly identified NEAT domains within this group are similar to Sa-IsdC and Ba-IsdC, they are likely to possess similar function. The IsdC Group includes domains encoded by other pathogens, such as *Bacillus cereus* protein4 and *Listeria monocytogenes* protein2, suggesting these pathogens may employ a NEAT-mediated heme-acquisition mechanism during infection that is fundamentally similar to that of *S. aureus* and *B. anthracis* [15]. This group also includes NEAT domains encoded by a number of coagulase-negative staphylococci. These species are normally non-pathogenic but can cause opportunistic infections including septicemia, suggesting that they may utilize NEAT domains to acquire heme from the host during an infection, or during normal growth on an iron source [62], [63]. Also, the IsdC Group contains IsdC-like domains encoded by many non-pathogenic, environmental bacteria. The ligands for these may be environmental porphyrin, which are abundant in decaying matter in soil.

**Figure 4:**
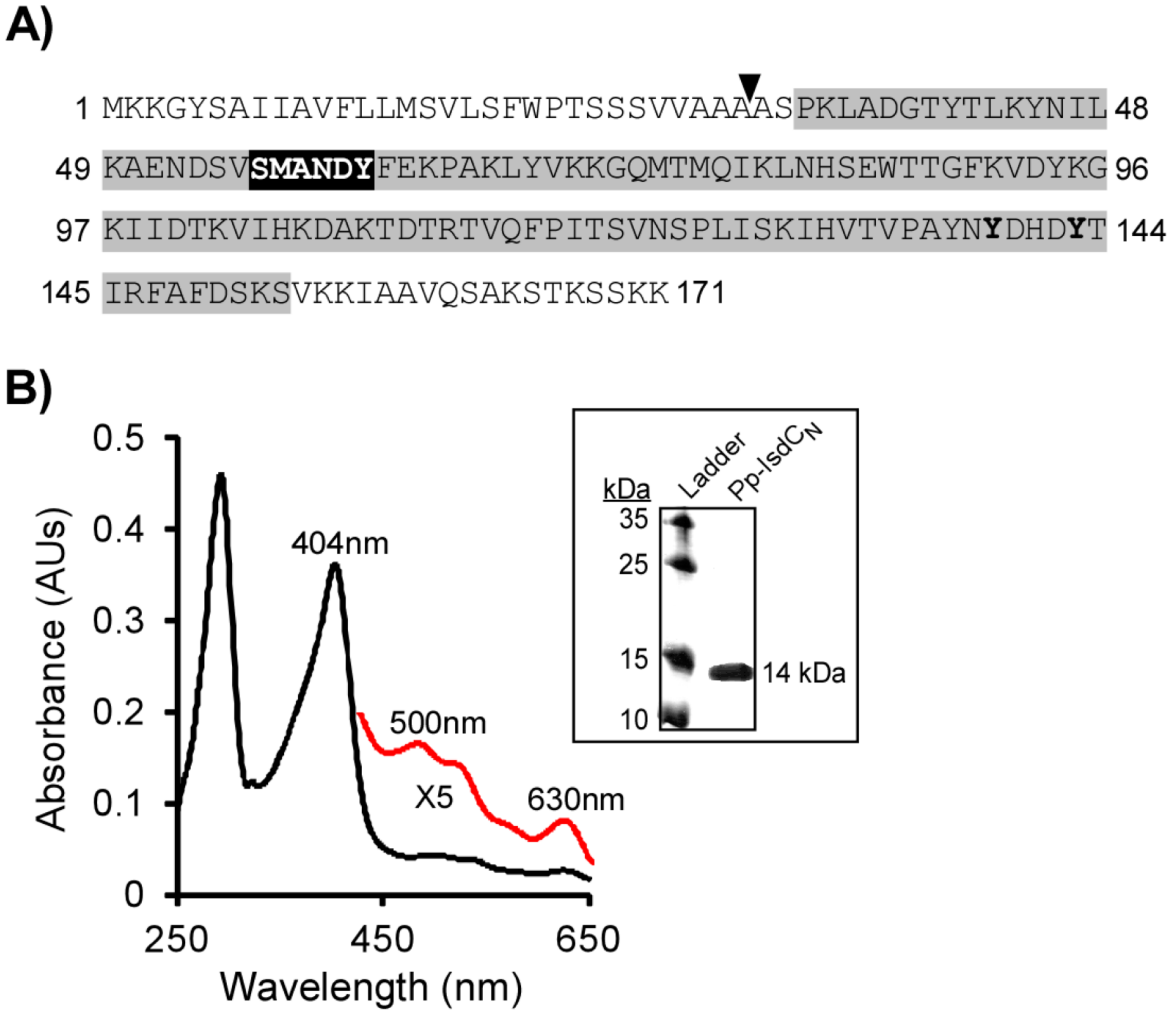
Amino acid sequence of Pp-IsdC and heme binding analysis. ***A***- Amino acid sequence of full-length Pp-IsdC. The signal cleavage site is indicated by the arrowhead, and the NEAT domain (Pp-IsdC_N_) is highlighted in grey. The 3_10_-helix is boxed in black, and the two tyrosine residues within the heme-binding signature sequence are bolded. ***B***- Spectral analysis of purified Pp-IsdC shows the presence of a Soret band at 404 nm, indicating that the protein can bind heme. The red spectra, magnified ×5, indicates Q bands at 500 and 630 nm, demonstrating that the heme bound by Pp-IsdC is oxidized (Fe^3+^) and is in a high-spin coordination [67]. ***Inset:*** SDS-PAGE of purified Pp-IsdC_N_ at 14 kDa.

To test whether an environmental organism (defined here as a bacterium that is not known to colonize or infect mammals) encodes a heme-binding NEAT domain, we chose to clone and purify an IsdC-like NEAT domain homologue from the nitrogen-fixing agricultural inoculant, *P. polymyxa* (protein4; Pp-IsdC_N_; Table S1). *P. polymyxa* is a known root-tip colonizer and symbiont of a wide range of crops, where it protects plants against bacterial pathogens, and against abiotic stress, and is found in the surrounding soil [55], [64], [65]. It is also proposed that by forming biofilms, *P. polymyxa* outcompetes pathogens for space and nutrients. These nutrients could include porphyrins (including heme) inside plant cells, or in the environment itself, such as in soil and decaying plant matter. Therefore, we employed *in vitro* biochemical studies using one of the 20 predicted *P. polymyxa* NEAT domains, to determine the conservation of porphyrin (heme) binding-function. The complete amino acid sequence of Pp-IsdC_N_ from *P. polymyxa*, with the NEAT domain highlighted in grey, is shown in Figure 4A. We cloned Pp-IsdC_N_ into a pGEX2TK vector and over-expressed the protein in *Escherichia coli* BL21 then purified the 14 kDa product to homogeneity using affinity chromatography (Figure 4B, inset).

Pp-IsdC_N_ was analyzed for the ability to bind endogenous heme synthesized by *E. coli* by detection of a strong absorbance (Soret band) at 400 nm, a commonly used absorbance profile that is specific to bound heme [2], [22], [37], [66], [67]. Purified Pp-IsdC_N_ produced a strong Soret band at 404 nm, indicative of co-purification with heme (Figure 4B). Additionally, as shown in Figure 4B (*red spectra*), characteristic Q-bands were detected at 500 and 630 nm, indicating oxidized heme was bound by the NEAT domain [67]. This suggests that, as with Ba-IsdC and Sa-IsdC, Pp-IsdC_N_ can bind oxidized heme [17], [32]. This is the first report of a NEAT domain expressed by a non-pathogenic bacterium possessing heme-binding function. Since *P. polymyxa* is exclusively environmental, the ability of Pp-IsdC_N_ to retain heme-binding activity suggests that the function of the NEAT domain is remarkably conserved, through the evolution of Gram-positive pathogens *vs.* soil-bacteria. As discussed, *P. polymyxa* has been shown to invade plant tissue as part of its natural relationship with plants [55]. Therefore, we postulate that soil organisms and plant symbionts expressing NEAT domain proteins may utilize them during growth to acquire porphyrins found in the environment, including chlorophyll (a highly similar porphyrin to heme). Chlorophyll binding by NEAT proteins expressed by these symbiotic organisms could be a process to acquire magnesium. Unfortunately, we could not biochemically determine specific chlorophyll-binding activity under our laboratory conditions. Future work could test this hypothesis, as several of these soil bacteria are highly desirable to the agricultural community, and supplementing with “nutrients” such as chlorophyll may increase the growth of these colonizing symbionts, to protect against plant pathogens and subsequently enhance crop growth.

#### The Clostridial Group

During the initial NEAT domain identification study in 2002, two putative NEAT proteins from *C. perfringens* were discovered, although no clostridial NEAT domains have been functionally characterized [15]. Our analysis identified 51 NEAT domains encoded by nine clostridial species, 46 of which had not been previously described. This Group formed a significant species-specific clade (Figure 2, magenta text). Proteins with members in this group ranged from those with a single NEAT within a full-length protein, to two proteins with seven NEAT domains within one polypeptide. Most contain an LPXTG sortase-A motif that would allow covalent anchoring to the cell wall (Table S1; [45]). This is similar to the *S. aureus* NEAT proteins IsdA, IsdB and IsdH that also contain the LPXTG motif [16], [17], [68]. The remaining seven clostridial proteins within this group, *Clostridium bartlettii* p2, *Clostridium botulinum* p1 and p2, *Clostridium butyricum* p1, *Clostridium celatum* p2, *Clostridium chauvoei* p2, and *C. perfringens* p2 are predicted to be cell surface-associated, however the exact cellular localization of these proteins unknown.

Within the Clostridial Group, five species are considered pathogens, with four of these, *C. chauvoei*, *C. novyi*, *C. perfringens* and *C. tetani*, being able to cause systemic infections that would allow exposure to the circulatory system and therefore to heme [69], [70], [71], [72]. This could explain why these anaerobic pathogens possess putative cell surface NEAT domains. *C. botulinum* is also a pathogen, however is mostly found within soil; its neurotoxins contaminate food supplies to cause the botulism disease [73], [74]. As discussed for the IsdC Group, *C. botulinum* may be exposed to heme, or similar porphyrins such as chlorophyll, during normal growth in the environment, and exploit them as a nutrient supply. The remaining species within the Clostridial Group are non-pathogenic gastrointestinal commensals [*C. bartlettii* and *C. butyricum* (also a probiotic)], and as such may use their NEAT domains to acquire heme from ingested foods in the gut as a source of iron [75], [76]. These hypotheses will require further testing to determine the biological significance of anaerobic clostridial bacteria expressing NEAT domains on their cell surface.

**Figure 5:**
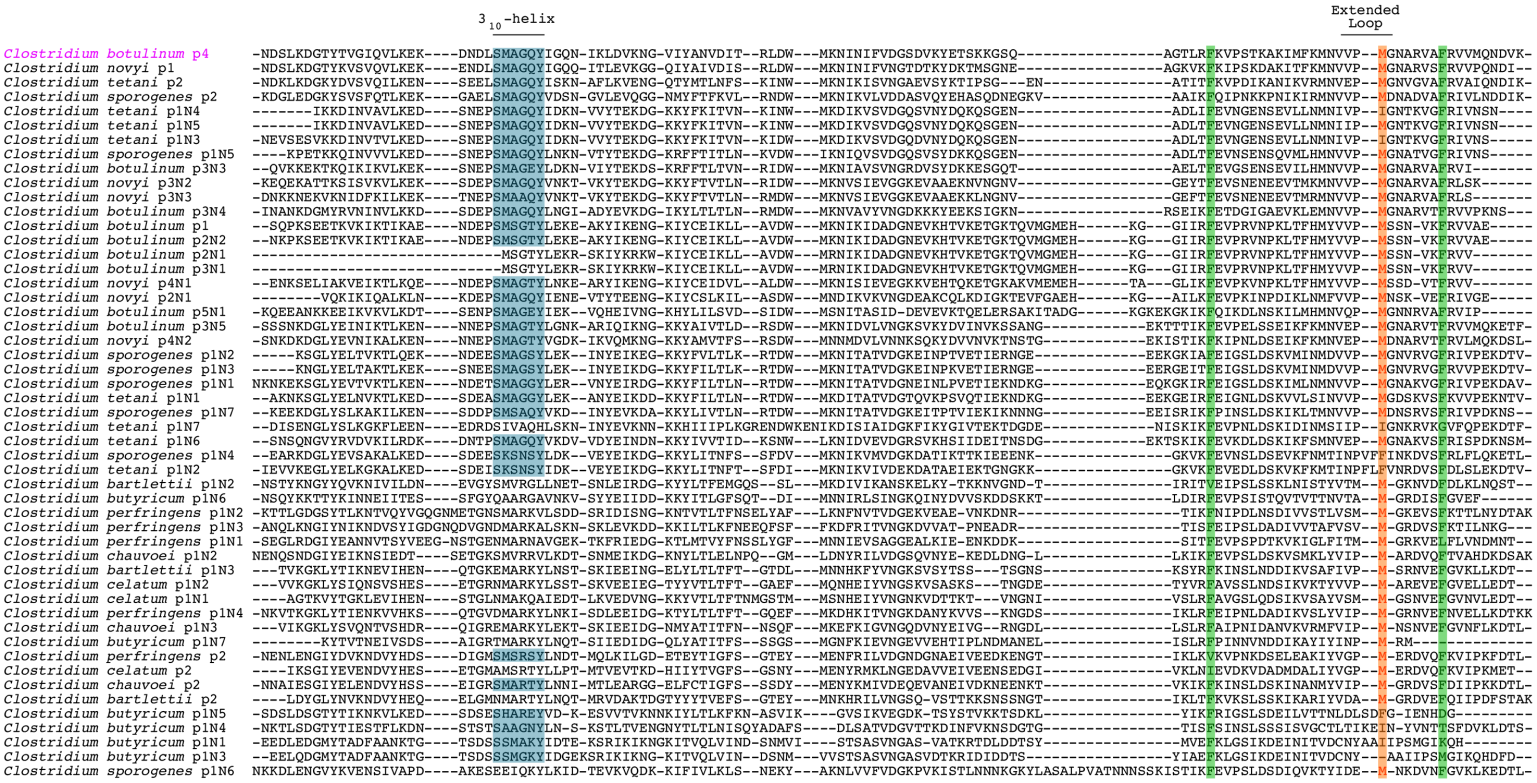
Alignment of Clostridial Group NEAT domains. The alignment was generated by ClustalX. The canonical 3_10_-helix sequence, SXXXXY, present in 33 of the 51 NEAT domains in this group, is highlighted in blue. The locations of two highly conserved phenylalanine residues are boxed in green. The methionine residue predicted to interact with heme (**Figure 6**) is highlighted in red and boxed in orange. The *C. botulinum* protein4, which was chosen for structural modeling analysis, is highlighted in magenta text.

An alignment of the Clostridial Group NEAT domains illustrates the conservation of the 3_10_-helix sequence (SXXXXY) in 33/51 of the clostridial NEAT domains, similar to the helix in the *B. anthracis* IsdX1 and IsdX2 heme-binding domains (Figure 1 and Figure 5; [22], [27]). A recent study analyzed each of the five NEAT domains of IsdX2, to determine redundant or NEAT-specific function [40]. Using biochemical and structural analysis, it was demonstrated that NEAT domains must possess an amino acid with an amide side chain (glutamine or asparagine) at the fourth position within the 3_10_-helix to actively scavenge heme from hemoglobin. Fourteen of 51 NEAT domains in the Clostridial Group that have this glutamine. In addition, 10 of these 14 NEAT domains are encoded by pathogens, suggesting there may be a conservation of NEAT heme binding and heme scavenging function, an activity not previously reported for clostridial pathogens. These data indicate that clostridial pathogens could be exposed to porphyrins during an infection, activating an IsdX1- or IsdX2-like process to acquire heme from hemoglobin.

In contrast, the heme-binding signature sequence (YXXXY) is absent from all NEAT domains within the Clostridial Group (Figure 5). Since the YXXXY sequence is essential for heme binding by *S. aureus* and *B. anthracis* NEAT domains, a different set of requirements for heme binding by clostridial NEAT domains may exist. This also likely explains the separate clustering of these domains from those of *S. aureus* and *B. anthracis* (Figure 2). We propose that one or both of two conserved phenylalanine residues within the clostridial NEAT domains may be able to replace the aromatic tyrosine iron-coordination bond, to bind the heme-iron atom (Figure 5, green highlights). In support of this concept, the *B. anthracis* NEAT-protein, Hal, has a phenylalanine residue in place of the second tyrosine in the heme-binding signature sequence, and is still able to bind heme [28]. Further, mutational analysis of the IsdA NEAT domain from *S. aureus* showed that substitution of the iron-axial ligand tyrosine to an alanine resulted in a histidine in the 3_10_-helix compensating by acting as the iron-axial ligand [37]. These data suggest that there is some flexibility in residue position and composition of NEAT functional regions, allowing for multiple scenarios for heme-binding function. A recent study utilized structural and bioinformatics data to predict possible axial ligands of heme-binding proteins. For heme found within hemoglobin, the top five residues with high relative frequency as axial ligands were cysteine, histidine, phenylalanine, methionine and tyrosine [77]. The position of the two highly conserved clostridial phenylalanines toward the carboxy-terminus of the NEAT domains, where the traditional YXXXY heme-binding sequence is located, may suggest a novel heme-binding capacity employed by clostridial NEAT domains (Figure 5). The concept of conserved NEAT domain structure, with altered sequence-specific heme coordination function, has been demonstrated for the IsdB heme receptor of *S. aureus* [43]. However, it is also possible that clostridial NEAT domains have an alternate role in anaerobic bacteria that has yet to be identified.

**Figure 6:**
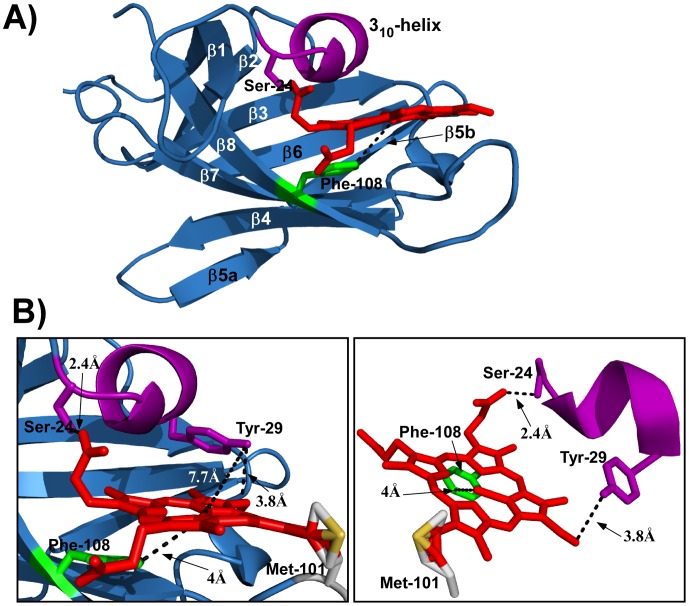
Model of *C. botulinum* p4 NEAT domain. SWISS-MODEL, Coot and PyMOL were used to generate a model of Cb-p4 coordinating heme. ***A***- The ribbon structure of Cb-p4 NEAT shows the eight β-strands in blue, and the 3_10_-helix in purple. The putative axial-ligand, Phe-108, is shown in green, with a possible coordination event from the Phe-108 side-chain to the iron-atom shown as a black dotted line. The modeled positioning of the heme (red) allowed the interaction between Ser-24 and the buried propionate heme group to be calculated (black dotted line). ***B***- Left panel is a close view of the heme-binding pocket, with possible coordination events demonstrated as black dotted lines. The Tyr-29 side-chain is shown in purple; Tyr-29 cannot bind heme-iron, but may undergo π-stacking interactions with the porphyrin ring. The Met-101 side-chain is in grey and the sulfur atom is in yellow. Met-101 is predicted to form multiple conformations, and may interact with heme. Right panel demonstrates four possible interaction events between the clostridial NEAT domain and heme (NEAT β-strands removed for clarity).

Taking into account our current knowledge of the relationship between NEAT sequence, structure and function, we sought to predict the structure of clostridial NEAT domains, and the location of the putative heme-iron coordinating residues. Predicting such a model would provide preliminary data needed to unravel the potentially unique heme-acquisition mechanism of clostridial NEAT domains. We employed comparative structural modeling of the NEAT domain of *C. botulinum* protein4 (Cb-p4) against the known structure of Ba-IsdX1. Figure 6A shows conservation of the NEAT immunoglobulin-like fold, comprised of the required eight β-strands and the 3_10_-helix. Phe-108, noted as a possible heme-iron coordinating residue, is located on the β8-strand at the position comparable to the second tyrosine in the conventional YXXXY heme-binding sequence (Figure 1). Using Coot modeling, superimposition of heme from Ba-IsdX1 into the Cb-p4 heme-binding pocket allowed a prediction of how heme could be coordinated within the heme-binding pocket, and also helped facilitate the identification of potential heme-coordinating residues. In Figure 6B (left panel), the distance between the farthest Phe-108 hydrogen and the heme-iron is predicted to be 4 Å. While this is not optimal for Fe^3+^ coordination, the iron atom was not included in the heme molecule in our Cb-p4 model. Therefore, the distance between Phe-108 and the iron could be reduced when the iron atom is present, to a distance that would allow Phe-108 to be a heme-iron axial ligand [17], [78], [79], [80]. Also, as determined during an *in silico* analysis, phenylalanine (as well as cysteine, histidine, methionine and tyrosine) can act as heme-axial ligands [77]. However, if Phe-108 is unable to coordinate heme-iron, there may be compensatory π-stacking interactions between this residue and Tyr-29 on the opposite side of the heme, as well as further interactions between other Cb-p4 residues and heme, as discussed below.

The 3_10_-helix residues of Cb-p4 are highly similar to those of the Ba-IsdX1 and Ba-IsdX2 heme-binding NEAT domains [22], [27], [36]. In Ba-IsdX1, Ser-24 H-bonds with the buried propionate group of heme, at a distance of 2.6 Å (Figure 1). In the model of Cb-p4, Ser-24 is proposed to H-bond with the same heme propionate group, at a distance of 2.4 Å. This interaction suggests that a heme-NEAT coordination event may occur at the 3_10_-helix (Figure 6B, left panel). Further, Tyr-29 of the 3_10_-helix, conserved in the conventional SXXXXY helix sequence, is in close proximity to a vinyl group of the heme molecule (Figure 6B, left panel), however vinyl groups cannot form H-bonds with hydroxyl groups. Therefore, as discussed, this tyrosine may instead undergo π-stacking interactions with the heme-ring, and coupled with Phe-108 π-stacking, could increase heme-NEAT affinity [36], [77].

A fourth possible NEAT-heme interaction site was detected between Met-101 and the second heme vinyl group (Figure 5, red highlighted residues; Figure 6B, left panel). To our knowledge, this interaction has not been described between a heme-binding protein and iron-porphyrin, and may be a clostridial-specific NEAT-heme coordination event. However, previous studies of the Shp NEAT protein from *S. pyogenes* revealed a bi-methionyl coordination event between the heme-iron and two methionine residues [15]. In our homology-modeled structure, the side chain of Met-101 was predicted to assume multiple conformations, one of which directly interacted with the heme vinyl in all conformations tested. Met-101, found on an extended loop between the β7- and β8-strands, could further increase the affinity between NEAT and heme, however this is only an hypothesis. This interaction may compensate for the lack of a comparable amino terminal tyrosine in the clostridial heme-binding sequence. Figure 6B (right panel) demonstrates the four possible interactions between the NEAT heme-binding pocket, and the heme porphyrin. Several hydrophobic residues (not shown) line the inside of the heme-binding pocket. These would increase heme-binding ability, since heme is a hydrophobic molecule [2], [78]. This hydrophobic environment is a fully conserved property of NEAT domains, and is formed by the β-barrel structure of the domain [15].

While the Cb-p4 structure is a model based on homology to IsdX1, it is encouraging that the conserved structure and fold of the NEAT domain is retained and the fundamental residues needed for heme binding are analogous to those already established. The non-heme binding NEAT domains of *S. aureus* (IsdB NEAT1 and IsdH NEAT1 and NEAT2) do not contain the canonical SXXXXY 3_10_-helix sequence (FYHYAS, YYHFFS and FYHYAS, respectively), nor the YXXXY heme-binding sequence (EEKYD, ETNYD, and HEDYD, respectively). Since they lack both functional regions, these domains cannot scavenge heme from hemoglobin, or bind heme [29], [41], [81]. Instead, they may serve as structural support for the heme-acquiring NEAT domains of the respective proteins. Further, NEAT2 of the IsdX2 protein from *B. anthracis* lacks the iron-coordinating tyrosine in the YXXXY sequence (YKQTH), and is unable to bind heme, yet it interacts with hemoglobin (SXXXXY sequence is conserved: SKMNTY) and may form a stabilizing contact for the other IsdX2 NEAT domains to extract heme from hemoglobin [27]. Therefore, the data obtained from previous studies, coupled with our clostridial NEAT domain model, increases the possibility that certain clostridial NEAT domains we identified possess the structural requirements needed to bind heme and/or hemoglobin, and acquire heme from hemoglobin [82].

Together, we have identified five possible interaction events that may allow clostridial NEAT domains to bind heme in a novel manner: i) Phe-108, which may provide an axial-ligand interaction with iron; ii) π-stacking interaction(s) of the aromatic side chain of Tyr-29 and Phe-108 with each side of the heme porphyrin; iii) Ser-24 in the 3_10_-helix that may H-bond with a propionate side-group of heme; iv) a possible interaction of Met-101 with heme; and v) overall hydrophobic interactions with the internal NEAT heme-binding pocket and heme. Also, as noted, IsdB of *S. aureus* possesses a unique heme-iron coordination requirement at the amino acid level, so the concept of a new heme-binding regime is not unprecedented [43]. Here, we present the first *in silico* analysis of possible clostridial NEAT-heme acquisition function. Future studies are needed to solve the crystal structure of Cb-p4 and to test the biochemical function(s) of the clostridial NEAT domains. Our data suggest that several clostridial species harbor NEAT domains that may act to acquire nutrients from the host by a novel heme-binding mechanism. Also, this is the first homology-modeling and *in silico* analysis of a NEAT-domain from an anaerobic pathogen since the initial identification of the two *C. perfringens* proteins [15].

### Overall NEAT sequence and function conservation

Thus far, we have provided evidence for possible groups of NEAT domains based on their *in vitro* function, which we elucidated from previous studies focusing on NEAT proteins from Gram-positive pathogens. However, as the phylogenetic tree shows, the majority of the 343 NEAT domains do not fit into a subgroup that contains characterized NEAT domains. Therefore, the overall amino acid sequence conservation in critical regions of the NEAT domain, as well as similarities and differences, are addressed here.

We have previously discussed the importance of the 3_10_-helix, a stretch of six amino acids that is canonically SXXXXY. The serine in this region interacts with a heme propionate group; therefore, it is an important determinant of heme binding function (Figure 1). Additionally, the tyrosine at the end of the 3_10_-helix can undergo π-stacking interactions with the heme ring, further increasing NEAT-heme coordination strength. We therefore sought to determine the overall conservation of the 3_10_-helix sequence, to possibly suggest which of the 343 NEAT domains could bind heme, if SXXXXY were such a determinant. Of all NEAT domains detected in our analysis, 245/343 (71%) have the SXXXXY sequence. Seven of these NEAT domains (2%) contain a one amino acid insertion within the 3_10_-helix, yet they retain the amino- and carboxy-terminal serine and tyrosine. Two of these NEAT domains (0.6%) harbor a single amino acid deletion within the helix. Thirty-nine NEAT domains (11.3%) possess the serine within the 3_10_-helix, however they have a different aromatic residue in place of the tyrosine (SXXXXF/H); of these, 38 contain a phenylalanine, and one has a histidine. These NEAT domains may have a somewhat different heme-binding environment, utilizing phenylalanine π-stacking, similar to what was proposed for the clostridial NEAT domains. Finally, 59 of the NEAT domains (17%) do not have the canonical SXXXXY sequence within the 3_10_-helix. This group includes the IsdB NEAT1 and IsdH NEAT1 and NEAT2 domains of *S. aureus*, which do not bind heme or acquire heme from hemoglobin. This indicates that the NEAT domains that do not have the serine or an aromatic residue at the terminus of the 3_10_-helix, do not function as heme acquisition modules, but instead might provide a stabilizing action for other NEAT domains within the same protein to bind heme [21], [29].

The slight extension of the β7-β8 loop that was observed in Cb-p4 (Figure 6) is predominant in members of the Clostridial Group. Perhaps the highly conserved methionine in an extended loop is a unique property that compensates for the lack of previously identified heme iron axial ligands. This hypothesis also requires further testing.

Analysis of the YXXXY heme-binding sequence revealed that approximately half of the predicted NEAT domains contain this critical motif (165/343, 48%). These 165 NEAT domains fell into three groups: i) has both tyrosines (48%), suggesting they are able to bind heme (*e.g.* Pp-p4, IsdC and BslK proteins of *B. anthracis* and *S. aureus*, IsdX1, IsdX2 NEAT1, NEAT3, NEAT4, and NEAT5, IsdA, IsdB NEAT2, and IsdH NEAT3) [22], [26], [27], [29], [43]; ii) retain the first tyrosine, but has another aromatic amino acid (31/343 or 9% histidine and 3/343 or 1% phenylalanine) in place of the second tyrosine (*e.g.* IsdX2 NEAT2 of *B. anthracis*, which is unable to bind heme); and iii) lacks both tyrosine residues. Many of the NEAT domains that belonged to the third group lack any noticeable heme-binding motifs, suggesting that either these NEAT domains play a structural role, as is the case with the non-heme binding NEAT domains of IsdB and IsdH of *S. aureus*, or there is an as yet unidentified heme-coordinating axial ligand(s) in these proteins [41]. Again, biochemical and structural work on select NEAT domains and the full-length NEAT proteins could further ascertain this.

Finally, the 343 NEAT domains analyzed are approximately the same length as previously characterized NEAT domains (average length 120 amino acids). The shortest NEAT domain was p1N1 of *Clostridium bartlettii* (89 amino acids). It did not cluster with the other clostridial NEAT domains, but rather it clusters near the IsdC group. The two longest NEAT domains identified are p1N2 of *Eggerthella* sp. and p1 of *Clostridium methylpentosum* (both 159 amino acids long). These two NEAT domains cluster close to each other, with high bootstrap scores, but they map at the terminus of the tree that had few groups with multiple family members. Neither have a SXXXXY or YXXXY motif. Thus, while the amino acid similarity between all NEAT domains and the lengths may vary, the importance of NEAT structure and function relationships is more related to secondary structure, specific amino acid residues within the 3_10_-helix, the heme-binding YXXXY sequence, and the overall hydrophobic nature of the heme-binding pocket.

## Conclusions

We identified 343 putative NEAT domains from 82 different bacterial species that were almost exclusively members of the Firmicutes. Perhaps surprisingly, no new members were discovered in metagenomic data sets from the Human Microbiome Project. This may indicate that in humans, NEAT domain proteins are generally restricted to pathogenic species. Protein parsimony analysis allowed clustering of predicted NEAT domains into known groups that included new members, and into new NEAT domain Groups that have yet to be functionally characterized. These results also allowed us to report: i) additional pathogenic organisms that encode putative NEAT domains that potentially act as heme-iron acquisition systems employed during infection; ii) the conservation of NEAT domains within the Firmicutes, including their presence in environmental and plant symbiotic bacteria; iii) the conservation of heme-binding function by a NEAT homologue encoded by a non-pathogenic environmental species; iv) comparative-modeling to demonstrate that NEAT structure may be conserved despite amino acid sequence differences; and v) altered structural requirements for heme binding by several divergent clostridial NEAT domains. Together, the data presented provide information describing 343 NEAT domains, 328 of which are new. Our data furthers the understanding of NEAT structure and function. As new bacterial genome sequences become available, more NEAT domains will be discovered. Work presented here will assist future research projects that target NEAT domains with anti-microbial molecules as a strategy to inhibit pathogens from accessing the rich heme-iron pool during infection.

## Materials and Methods

### Identification of putative NEAT domains

NEAT domain-containing protein sequences were identified by protein-protein BLAST (blastp) against the NCBI Non-redundant protein sequences (nr) database (update 11/1/2013) using the *S. aureus* IsdC protein sequence as the query (gi: 285816811). It is a representative member of the Conserved Domains family cd06920, NEAr Transport domain, and pfam05031 [15]. The blastp search parameters were the default as defined [83]. The search returned over 3,500 bacterial protein sequences annotated as containing cd0692, however many of these were identical sequences from multiple strains of a species, so a single representative of each putative NEAT-containing protein was selected for further analysis. Next, each full-length protein was analyzed using Pfam v26 [84], [85] to determine the number of NEAT domains within the protein sequence and the exact residues constituting each NEAT domain. NEAT domains with Pfam e-scores less than e^−11^ were removed, resulting in a final count of 185 unique proteins, encoding 343 NEAT domains. Protein subcellular localization was predicted using PSORTb (version 3.0.2; [86]).

We also performed a blastp search of the human filtered, translated whole genome shotgun sequences corresponding to each body site sampled and sequenced for the Human Microbiome Project (HMGI, www.hmpdacc.org). A separate blast database was created for each of the 18 body sites then was searched by blastp 2.2.28+ using IsdC as the query, as before.

In this study, NEAT proteins for each species are numbered p1, p2, p3 etc. For multiple NEAT domains in a single protein, the NEAT domains are numbered N1, N2, N3 etc., from the amino-terminus to the carboxy-terminus. If the protein and NEAT domains have been previously characterized, their published names are used (see Table S1 for proteins and accession numbers).

### Phylogenetic analysis

Bootstrapped phylogenetic trees of the amino acid sequences of the known and predicted NEAT domains were created using the Protpars protein parsimony algorithm within the PHYLIP package (version 3.69, [87], [88]). The NEAT domain sequences were aligned using ClustalX with iteration (version 2.1, [83], [89]), then the sequences were bootstrapped by running 100 replicates using seqboot. The datasets were passed to protpars then consense was used to generate a majority rule unrooted tree. The final trees were displayed using MEGA 5.2.2 [90].

### Identification and cloning of *Paenibacillus polymyxa* IsdC NEAT

*P. polymyxa* strain ATCC 842 was obtained from ATCC (Manassas, VA, USA). The seed bacteria were inoculated into 6 mL lysogeny broth (LB) and grown for 48 hr at 30°C. Chromosomal DNA was isolated from cultures using the E.Z.N.A bacterial DNA kit (Omega Bio-Tek, Georgia, USA). The genome of *P. polymyxa* ATCC 842 was recently sequenced [53], and whole genome shotgun contigs are deposited as accession number AFOX01000036. Using the Pathosystems Resource Integration Center (PATRIC, www.patricbrc.org/portal/portal/patric/), we determined that the putative *P. polymyxa isdC* (*Pp-isdC*) gene was located on contig 36 [91]. Blastn [92] analysis using the nucleotide sequence of *Pp-isdC* from the E681 strain (used in the phylogenetic analysis) as the template, identified 32 nucleotide mismatches between E861 and ATCC 842 *Pp-isdC*; however at an amino acid level, there were only eight amino acid mismatches, none of which were located within the NEAT domain (Figure S1). We designed forward *Pp-isdC_NEAT_-Bam* HI ( *5′-*GTT-CGG-ATC-CCC-AAA-ATT-GGC-GGA-TGG-TAC-*3′* ) and reverse primers *Pp-isdC_NEAT_-Eco* RI ( *5′-*GCT-CGA-ATT-C **AA**-**T**GC-TTT-TGG-AAT-CAA-AAG-CGA-AGC-G-*3′* ) that allowed the NEAT domain of *Pp-isdC* (*Pp-IsdC_N_*) to be PCR-amplified from the ATCC 842 strain, using *Pfu* AD turbo polymerase (Agilent, Santa Clara, CA, USA). The reverse primer had an artificial stop codon (**UAA**) inserted before the *Eco* RI restriction site, as only the nucleotides encoding the NEAT domain were PCR-amplified, and the *Pp-isdC* stop codon was not cloned. The resulting PCR product was digested using *Bam* HI and *Eco* RI restriction enzymes (New England Biolabs, Ipswich, MA, USA) and ligated between the *Bam* HI/*Eco* RI sites of the pGEX2TK vector [27] to create a protein fusion to glutathione *S*-transferase (GST). The recombinant plasmid was then transformed into *E. coli* DH5α, plasmid DNA was recovered, the sequence of the insert was confirmed, and pGEX2TK::*gst*-*Pp-isdC_N_* was transformed into *E. coli* BL21 for protein expression.

### Pp-IsdC_N_ protein expression, purification and heme binding assay

The *E. coli* BL21 strain harboring pGEX2TK::*gst-Pp-isdC_N_* was grown in LB broth supplemented with 50 µg/mL ampicillin and 25 µg/mL kanamycin at 37°C. Pp-IsdC_N_ was expressed using 1.5 mM isopropyl β-D-thiogalactopyranoside (Sigma, St Louis, MO, USA) induction for three hours at 37°C. Cells were centrifuged (6,000×*g*) and resuspended in 50 mM Tris-HCl, pH 7.0. Bacteria were lysed using a French press and centrifuged at 14,000×*g*, and supernatants were applied to glutathione-Sepharose resin (GE Healthcare, Humble, TX, USA). After one 30 mL wash with Tris-HCl buffer (50 mM, pH 7.0), Pp-IsdC_N_ was eluted off the column after an overnight incubation with 50 units of thrombin (Calbiochem, Rockland, MA, USA) at room temperature to isolate Pp-IsdC_N_. Thrombin was removed using aminobenzamidine resin (Sigma, St Louis, MO, USA). Five µg purified, recombinant Pp-IsdC_N_ was analyzed by SDS-PAGE Coomassie stain (Figure 4B, inset).

To measure heme binding, spectroscopic scans were taken of the purified Pp-IsdC_N_ eluates from 250 to 650 nm under visible light using a Beckman Coulter DU800 spectrophotometer (VWR, Radnor, PA, USA).

### Modeling of *Clostridium botulinum* protein4 NEAT structure

The amino acid sequence of the NEAT domain of protein4 from *C. botulinum* (Cb-p4) was modeled using SWISS-MODEL [93], [94] in both automated and target-template modes using the *B. anthracis* IsdX1 chain A (heme-bound) as the target structure (PDB code: 3ISK; [36]). The best model obtained was for IsdX1 bound to heme was identified by the automated HHblits Hidden Markov method. The sequences were 23% identical and 33% similar over 96% of the target length. The QMEAN4 score was −3.12 and the normalized QMEAN4 score was 0.7; these values are indicative of a medium quality model [95]. Coot (version 0.6.2; [96]) was used to superimpose the heme ligand from IsdX1 chain A into the predicted correct conformation in the heme-binding pocket of Cb-p4, taking into account amino acids different between IsdX1 and Cb-p4. PyMOL (version 1.5; [97]) was used to produce the homology-modeled figures of Cb-p4, and to calculate H-bond distances.

## Supporting Information

Figure S1 **Alignment of IsdC gene (A) and protein (B) sequences from** ***Paenibacillus polymyxa*** **from strains ATCC 842 and E681.** The NEAT domains are highlighted in grey. Nucleotide mismatches are highlighted in black in (A). In (B), asterisks indicate identical residues and colons indicated conserved residues; the 3_10_-helix sequence is designated by the horizontal line and the conserved phenylalanine residues are indicated in bold; the red arrowhead points to the single non-conserved amino acid residue.
(TIFF)

Table S1 **List of 185 putative NEAT domain-containing proteins identified by blastp using** ***Staphylococcus aureus*** **IsdC (gi: 285816811) as the query.** Protein Name, GenBank Accession Number, Amino Acid Length, Number of NEAT domains per protein, Additional Features and Predicted Cellular Localization, based on PSORTb prediction, are reported. Abbreviations: LRR, leucine rich repeat; FMN flavin mononucleotide; SLH, surface layer homology; FIVAR, found in various architectures domain; LPXTG, cell wall anchor domain; YSIRK, gram-positive signal peptide motif; fn3, fibronectin type 3 domain.
(PDF)

Table S2 **List of organisms carrying genes predicted to encode NEAT domain proteins arranged by phylum, class, order, family, genus and species.**
(PDF)
