# Supplementary figures and images for: Molecular and Evolutionary Analysis of NEAr-Iron Transporter (NEAT) Domains
Source: PLoS One. 2014 Aug 25;9(8):e104794. doi: 10.1371/journal.pone.0104794 (PMC4143258; doi:10.1371/journal.pone.0104794)

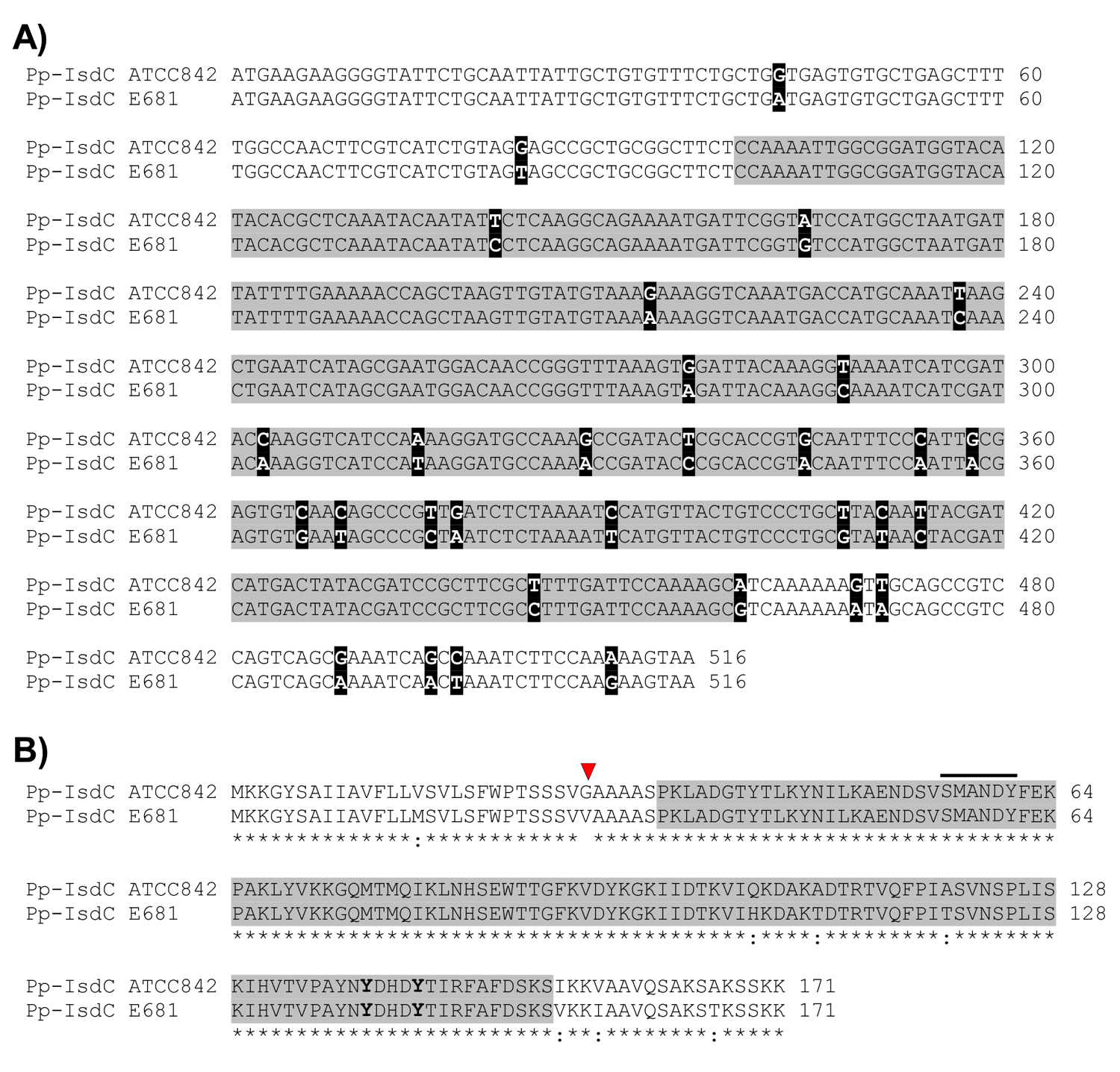

Supplement: Figure S1 — Alignment of IsdC gene (A) and protein (B) sequences from Paenibacillus polymyxa from strains ATCC 842 and E681. The NEAT domains are highlighted in grey. Nucleotide mismatches are highlighted in black in (A). In (B), asterisks indicate identical residues and colons indicated conserved residues; the 310-helix sequence is designated by the horizontal line and the conserved phenylalanine residues are indicated in bold; the red arrowhead points to the single non-conserved amino acid residue. (TIFF) [file pone.0104794.s001.tiff]
