# Supplementary material for: Molecular and Evolutionary Analysis of NEAr-Iron Transporter (NEAT) Domains
Source: PLoS One. 2014 Aug 25;9(8):e104794. doi: 10.1371/journal.pone.0104794 (PMC4143258; doi:10.1371/journal.pone.0104794)
Supplement: Table S1 — List of 185 putative NEAT domain-containing proteins identified by blastp using Staphylococcus aureus IsdC (gi: 285816811) as the query. Protein Name, GenBank Accession Number, Amino Acid Length, Number of NEAT domains per protein, Additional Features and Predicted Cellular Localization, based on PSORTb prediction, are reported. Abbreviations: LRR, leucine rich repeat; FMN flavin mononucleotide; SLH, surface layer homology; FIVAR, found in various architectures domain; LPXTG, cell wall anchor domain; YSIRK, gram-positive signal peptide motif; fn3, fibronectin type 3 domain. (PDF) [file pone.0104794.s002.pdf]

Table S1. Putative NEAT proteins

Genus and *species*	Protein Name	Accession #	Amino Acid Length	# NEAT Domains	Other Features	Predicted Localization	Reference
Anaerostipes hadrus	Protein 1	WP_009203222	968	1	Domain of unknown function (DUF1533); Uncharacterized sugar binding domain; 1 LRR; fn3 domain.	Multiple Localization Sites (secreted/cell wall)	This study
Anaerostipes hadrus	Protein 2	WP_009203221	740	1	FMN-binding domain; fn3 domain	Multiple Localization Sites (secreted/cell wall)	This study
Anaerostipes hadrus	Protein 3	WP_009203220	512	1	None	Multiple Localization Sites (secreted/cell wall)	This study
Bacillus anthracis	Hal	AAT29649	1070	1	6 LRR (2 copies of each)	Multiple Localization Sites (secreted/cell wall)	[28]
Bacillus anthracis	BslK	AAT30191	344	1	3 SLH domains	Cell Surface	[26]
Bacillus anthracis	IsdX1	AAT33910	152	1	None	Secreted	[22, 33, 36, 59]
Bacillus anthracis	IsdX2	AAT35435	885	5	None	Secreted	[22, 27, 33, 59]
Bacillus anthracis	IsdC	AAT33911	237	1	None	Cell wall anchored (sortase motif)	[22, 32]
Bacillus anthracis	Protein 6	ZP_00390931	427	1	3 LRR (2 copies each)	Cell Surface	This study
Bacillus cereus	Protein 1	ACO25697	153	1	None	Secreted	This study

Table S1. Putative NEAT proteins

Genus and *species*	Protein Name	Accession #	Amino Acid Length	# NEAT Domains	Other Features	Predicted Localization	Reference
Bacillus cereus	IlsA	ACO30186	1186	1	4 LRR (2 copies each)	Cell Surface	[24]
Bacillus cereus	Protein 3	ACO30570	925	5	1 repeat of unknown function (DUF1388)	Cell Surface/Secreted	This study
Bacillus cereus	Protein 4	ACO31200	233	1	None	Cell Surface	This study
Bacillus clausii	Protein 1	BAD65956	798	4	None	Cell Surface	This study
Bacillus clausii	Protein 2	BAD65957	230	1	None	Cell Surface	This study
Bacillus halodurans	Protein 1	BAB07017	1071	5	None	Cell Surface	This study
Bacillus halodurans	Protein 2	NP_244165	221	1	None	Secreted	This study
Bacillus mycoides	Protein 1	EEL97292	1066	6	None	Cell Surface/Secreted	This study
Bacillus mycoides	Protein 2	EEL97293	152	1	None	Secreted	This study
Bacillus mycoides	Protein 3	EEL97294	244	1	None	Cell Surface	This study

Table S1. Putative NEAT proteins

Genus and *species*	Protein Name	Accession #	Amino Acid Length	# NEAT Domains	Other Features	Predicted Localization	Reference
Bacillus mycoides	Protein 4	EEM00228	760	1	3 LRR (2 copies each); 3 SLH domains	Cell Surface	This study
Bacillus mycoides	Protein 5	EEM00516	341	1	3 SLH domains	Cell Surface	This study
Bacillus pumilus	Protein 1	EDW21502	605	3	None	Cell Surface/Secreted	This study
Bacillus sp. GeD10	Protein 1	CCW04153	760	1	4 copy LRRs; 3 SLH domains	Cell Wall (SLH)	This study
Bacillus sp. GeD10	Protein 2	CCW07528	970	1	5 copy LRRs	Cell Surface	This study
Bacillus sp. GeD10	Protein 3	CCW08180	942	5	None	Cell Surface	This study
Bacillus sp. GeD10	Protein 4	CCW08178	152	1	None	Cell Surface	This study
Bacillus sp. GeD10	Protein 5	CCW08178	237	1	None	Cell Surface	This study
Bacillus thuringiensis	Protein 1	ADH05290	971	1	5 LRR (2 copies each); 2 LRR adjacent	Cell Surface	This study
Bacillus thuringiensis	Protein 2	ADH05990	760	1	4 LRR (2 copies per LRR); 3 SLH domains	Cell Surface	This study

Table S1. Putative NEAT proteins

Genus and *species*	Protein Name	Accession #	Amino Acid Length	# NEAT Domains	Other Features	Predicted Localization	Reference
Bacillus thuringiensis	Protein 3	ADH09001	935	5	None	Secreted	This study
Bacillus thuringiensis	Protein 4	ADH09002	152	1	None	Secreted	This study
Bacillus thuringiensis	Protein 5	ADH09003	237	1	None	Cell Surface	This study
Bacillus weihenstephanensis	Protein 1	ABY41733	1011	1	3 LRR (2 copies each); 2 LRR adjacent	Cell Surface	This study
Bacillus weihenstephanensis	Protein 2	ABY42262	343	1	3 SLH domains	Cell Surface	This study
Bacillus weihenstephanensis	Protein 3	ABY45534	1147	6	None	Cell Surface/Secreted	This study
Bacillus weihenstephanensis	Protein 4	ABY45535	152	1	None	Secreted	This study
Bacillus weihenstephanensis	Protein 5	ABY45536	236	1	None	Cell Surface	This study
Carnobacterium maltaromaticum	Protein 1	YP_006993791	216	1	None	Cell Surface	This study
Carnobacterium maltaromaticum	Protein 2	YP_006993790	713	4	None	Cell Surface	This study

Table S1. Putative NEAT proteins

Genus and *species*	Protein Name	Accession #	Amino Acid Length	# NEAT Domains	Other Features	Predicted Localization	Reference
Carnobacterium maltaromaticum	Protein 3	WP_010054623	707	4	None	Cell Surface	This study
Clostridium bartlettii	Protein 1	EDQ96013	592	3	None	Cell Wall (LPXTG anchored)	This study
Clostridium bartlettii	Protein 2	EDQ96014	283	1	None	Secreted	This study
Clostridium botulinum	Protein 1	AEB75283	325	1	None	Secreted	This study
Clostridium botulinum	Protein 2	AEB75284	381	2	None	Secreted	This study
Clostridium botulinum	Protein 3	AEB75285	891	5	None	Cell Wall (LPXTG anchored)	This study
Clostridium botulinum	Protein 4	AEB75286	233	1	None	Cell Wall (LPXTG anchored)	This study
Clostridium botulinum	Protein 5	AEB75300	1742	3	3 LRR (2 copies each)	Cell Wall (LPXTG anchored)	This study
Clostridium butyricum	Protein 1	EDT75540	1380	7	None	Cell Surface	This study
Clostridium celatum	Protein 1	WP_005211457	686	2	None	Cell Wall (LPXTG anchored)	This study

Table S1. Putative NEAT proteins

Genus and *species*	Protein Name	Accession #	Amino Acid Length	# NEAT Domains	Other Features	Predicted Localization	Reference
Clostridium celatum	Protein 2	WP_005211455	224	1	None	Multiple Localization Sites (secreted/cell wall)	This study
Clostridium chauvoei	Protein 1	CDG02635	1109	3	Domain of unknown function (DUF1533); 6 copy LRR; 2 copy LRR	Cell Wall (LPXTG anchored)	This study
Clostridium chauvoei	Protein 2	CDG02634	209	1	None	Multiple Localization Sites (secreted/cell wall)	This study
Clostridium methylpentosum	Protein 1	EEG30543	1593	1	4 FIVAR (uncharacterized sugar binding domain)	Cell Surface	This study
Clostridium nexile	Protein 1	EEA81527	549	1	None	Cell Wall (LPXTG anchored)	This study
Clostridium novyi	Protein 1	ABK60523	237	1	None	Cell Wall (LPXTG anchored)	This study
Clostridium novyi	Protein 2	ABK61289	906	2	1 LRR 1; 1 LRR 4 (2 copies)	Cell Wall (LPXTG anchored)	This study
Clostridium novyi	Protein 3	ABK61706	1675	3	3 DUF1533 (protein of unknown function); LRR 4 (2 copies each)	Cell Wall (LPXTG anchored)	This study
Clostridium novyi	Protein 4	ABK61993	467	2	None	Cell Wall (LPXTG anchored)	This study
Clostridium perfringens	Protein 1	ABG82492	743	4	None	Cell Wall (LPXTG anchored)	This study

Table S1. Putative NEAT proteins

Genus and *species*	Protein Name	Accession #	Amino Acid Length	# NEAT Domains	Other Features	Predicted Localization	Reference
Clostridium perfringens	Protein 2	ABG83763	215	1	None	Secreted	This study
Clostridium ramosum	Protein 1	EDS19586	507	3	None	Secreted	This study
Clostridium ramosum	Protein 2	EDS19593	1115	1	None	Secreted	This study
Clostridium spiroforme	Protein 1	EDS75668	508	3	None	Secreted	This study
Clostridium sporogenes	Protein 1	EDU37512	1227	7	None	Cell Wall (LPXTG anchored)	This study
Clostridium sporogenes	Protein 2	EDU37517	228	1	None	Cell Wall (LPXTG anchored)	This study
Clostridium tetani	Protein 1	AAO35763	1206	7	None	Cell Wall (LPXTG anchored)	This study
Clostridium tetani	Protein 2	AAO35764	294	1	None	Cell Wall (LPXTG anchored)	This study
Cohnella laeviribosi	Protein 1	WP_019006629	773	3	3 SLH domains	Cell Wall (SLH)	This study
Cohnella laeviribosi	Protein 2	WP_019006628	383	1	None	Cell Surface	This study

Table S1. Putative NEAT proteins

Genus and *species*	Protein Name	Accession #	Amino Acid Length	# NEAT Domains	Other Features	Predicted Localization	Reference
Cohnella laeviribosi	Protein 3	WP_019005992	813	1	3 SLH domains	Cell Wall (SLH)	This study
Coprobacillus sp. D7	Protein 1	EEO31145	1115	1	None	Secreted	This study
Coprobacillus sp. D7	Protein 2	EEO31151	506	3	None	Secreted	This study
Eggerthella sp. CAG:298	Protein 1	CDD59582	2222	2	None	Cell Wall (LPXTG anchored)	This study
Eubacterium yurii	Protein 1	EFM38371	427	1	3 SLH domains	Cell Surface	This study
Lachnospiraceae bacterium	Protein 1	EGG82294	532	1	None	Secreted	This study
Lactobacillus acidipiscis	Protein 1	WP_010495489	274	1	None	Multiple Localization Sites (secreted/cell wall)	This study
Lactobacillus brevis	Protein 1	ABJ63839	245	1	None	Secreted	This study
Lactobacillus brevis	Protein 2	ABJ63841	256	1	None	Secreted	This study
Lactobacillus brevis	Protein 3	EEI71315	160	1	None	Secreted	This study

Table S1. Putative NEAT proteins

Genus and *species*	Protein Name	Accession #	Amino Acid Length	# NEAT Domains	Other Features	Predicted Localization	Reference
Lactobacillus buchneri	Protein 1	EEI19753	280	1	None	Secreted	This study
Lactobacillus coryniformis	Protein 1	ZP_08478597	1317	5	None	Multiple Localization Sites (secreted/cell wall)	This study
Lactobacillus coryniformis	Protein 2	ZP_08572872	241	1	None	Secreted	This study
Lactobacillus coryniformis	Protein 3	ZP_08572873	695	2	None	Multiple Localization Sites (secreted/cell wall)	This study
Lactobacillus crispatus	Protein 1	EEU20208	239	1	None	Secreted	This study
Lactobacillus equicursoris	Protein 1	WP_008461456	262	1	None	Cell Surface	This study
Lactobacillus fermentum	Protein 1	EEX24773	262	1	None	Secreted	This study
Lactobacillus fermentum	Protein 2	EEX24774	621	1	None	Multiple Localization Sites (secreted/cell wall)	This study
Lactobacillus gastricus	Protein 1	WP_007122121	250	1	None	Cell Surface	This study
Lactobacillus gigeriorum	Protein 1	WP_008472986	272	1	None	Cell Surface	This study

Table S1. Putative NEAT proteins

Genus and *species*	Protein Name	Accession #	Amino Acid Length	# NEAT Domains	Other Features	Predicted Localization	Reference
Lactobacillus hilgardii	Protein 1	EEI25800	280	1	None	Secreted	This study
Lactobacillus mali	Protein 1	WP_010078176	285	1	None	Multiple Localization Sites (secreted/cell wall)	This study
Lactobacillus otakiensis	Protein 1	GAD17494	260	1	None	Multiple Localization Sites (secreted/cell wall)	This study
Lactobacillus parabrevis	Protein 1	WP_020088261	262	1	None	Cell Surface	This study
Lactobacillus parafarraginis	Protein 1	WP_008213376	276	1	None	Cell Surface	This study
Lactobacillus pasteurii	Protein 1	WP_009559431	267	1	None	Cell Surface	This study
Leuconostoc argentinum	Protein 1	ZP_08230679	267	1	None	Secreted	This study
Leuconostoc citreum	Protein 1	WP_004903428	245	1	None	Cell Surface	This study
Leuconostoc kimchii	Protein 1	ADG39491	245	1	None	Secreted	This study
Leuconostoc pseudomesenteroides	Protein 1	WP_010279598	246	1	None	Cell Surface	This study

Table S1. Putative NEAT proteins

Genus and *species*	Protein Name	Accession #	Amino Acid Length	# NEAT Domains	Other Features	Predicted Localization	Reference
Listeria innocua	Protein 1	EFR93130	573	3	None	Multiple Localization Sites (secreted/cell wall)	This study
Listeria innocua	Protein 2	EFR89979	208	1	None	Secreted	This study
Listeria ivanovii	Protein 1	EFR96098	195	1	None	Secreted	This study
Listeria ivanovii	Protein 2	EFR96102	447	2	None	Multiple Localization Sites (secreted/cell wall)	This study
Listeria marthii	Protein 1	EFR86991	205	1	None	Secreted	This study
Listeria monocytogenes	Protein 1	ADB69135	569	3	None	Multiple Localization Sites (secreted/cell wall)	This study
Listeria monocytogenes	Protein 2	YP_003414498	207	1	None	Multiple Localization Sites (secreted/cell wall)	This study
Listeria monocytogenes	Protein 3	ZP_05295259	180	1	None	Secreted	This study
Listeria monocytogenes	Protein 4	ZP_05296258	279	1	None	Multiple Localization Sites (secreted/cell wall)	This study
Listeria monocytogenes	Protein 5	ZP_05296258	480	2	None	Multiple Localization Sites (secreted/cell wall)	This study

Table S1. Putative NEAT proteins

Genus and *species*	Protein Name	Accession #	Amino Acid Length	# NEAT Domains	Other Features	Predicted Localization	Reference
Listeria seeligeri	Protein 1	EFR99345	207	1	None	Secreted	This study
Listeria seeligeri	Protein 2	EFR99352	446	2	None	Multiple Localization Sites (secreted/cell wall)	This study
Listeria seeligeri	Protein 3	YP_003465398	568	3	None	Multiple Localization Sites (secreted/cell wall)	This study
Listeria welshimeri	Protein 1	CAK21620	574	3	None	Multiple Localization Sites (secreted/cell wall)	This study
Listeria welshimeri	Protein 2	CAK21621	208	1	None	Secreted	This study
Lysinibacillus sphaericus	Protein 1	ACA38872	213	1	None	Multiple Localization Sites (secreted/cell wall)	This study
Lysinibacillus sphaericus	Protein 2	ACA38873	474	2	2 SLH domains	Cell Surface	This study
Lysinibacillus sphaericus	Protein 3	ACA38874	429	1	None	Cell Surface	This study
Lysinibacillus sphaericus	Protein 4	YP_001697002	213	1	None	Multiple Localization Sites (secreted/cell wall)	This study
Mollicutes bacterium D7	Protein 1	ZP_04565790	1115	1	None	Secreted	This study

Table S1. Putative NEAT proteins

Genus and *species*	Protein Name	Accession #	Amino Acid Length	# NEAT Domains	Other Features	Predicted Localization	Reference
Mollicutes bacterium D7	Protein 2	ZP_04565796	506	3	None	Secreted	This study
Paenibacillus daejeonensis	Protein 1	WP_020618812	356	1	None	Cell Wall (LPXTG anchored)	This study
Paenibacillus larvae	Protein 1	EFX45138	232	1	None	Multiple Localization Sites (secreted/cell wall)	This study
Paenibacillus larvae	Protein 2	EFX45139	452	2	None	Multiple Localization Sites (secreted/cell wall)	This study
Paenibacillus massiliensis	Protein 1	WP_018887299	1389	7	None	Cell Wall (LPXTG anchored)	This study
Paenibacillus massiliensis	Protein 2	WP_018887298	159	1	None	Multiple Localization Sites (secreted/cell wall)	This study
Paenibacillus massiliensis	Protein 3	WP_018887297	281	1	None	Multiple Localization Sites (secreted/cell wall)	This study
Paenibacillus peoriae	Protein 1	WP_010344678	1332	7	None	Cell Surface	This study
Paenibacillus peoriae	Protein 2	WP_010344677	167	1	None	Cell Surface	This study
Paenibacillus peoriae	Protein 3	WP_010344676	155	1	None	Cell Surface	This study

Table S1. Putative NEAT proteins

Genus and *species*	Protein Name	Accession #	Amino Acid Length	# NEAT Domains	Other Features	Predicted Localization	Reference
Paenibacillus peoriae	Protein 4	WP_010344675	171	1	None	Cell Surface	This study
Paenibacillus polymyxa	Protein 1	ADM71815	1065	7	None	Multiple Localization Sites (secreted/cell wall)	This study
Paenibacillus polymyxa	Protein 2	ADM71816	165	1	None	Secreted	This study
Paenibacillus polymyxa	Protein 3	ADM71817	155	1	None	Secreted	This study
Paenibacillus polymyxa	Protein 4	ADM71818	171	1	None	Multiple Localization Sites (secreted/cell wall)	This study
Paenibacillus polymyxa	Protein 5	ADO58493	1455	10	None	Multiple Localization Sites (secreted/cell wall)	This study
Paenibacillus sp. Aloe-11	Protein 1	WP_007432134	395	1	None	Multiple Localization Sites (secreted/cell wall)	This study
Paenibacillus sp. Aloe-11	Protein 2	WP_007432133	230	1	None	Cell Surface	This study
Paenibacillus sp. Aloe-11	Protein 3	WP_007432132	795	4	None	Cell Surface	This study
Paenibacillus sp. Aloe-11	Protein 4	WP_007432137	171	1	None	Cell Surface	This study

Table S1. Putative NEAT proteins

Genus and *species*	Protein Name	Accession #	Amino Acid Length	# NEAT Domains	Other Features	Predicted Localization	Reference
Paenibacillus sp. Aloe-11	Protein 5	WP_007432135	167	1	None	Multiple Localization Sites (secreted/cell wall)	This study
Paenibacillus sp. JDR-2	Protein 1	ACS99470	348	1	None	Multiple Localization Sites (secreted/cell wall)	This study
Paenibacillus sp. JDR-2	Protein 2	ACS99471	912	1	3 SLH domains	Cell Surface	This study
Paenibacillus sp. JDR-2	Protein 3	ACT00400	1149	2	3 SLH domains	Cell Surface	This study
Paenibacillus sp. JDR-2	Protein 4	ACT00403	331	1	None	Multiple Localization Sites (secreted/cell wall)	This study
Peptostreptococcus anaerobius	Protein 1	EFD05272	1844	1	3 Putative cell wall binding repeat2	Multiple Localization Sites (secreted/cell wall)	This study
Ruminococcus gnavus	Protein 1	EDN76872	445	1	None	Secreted	This study
Solibacillus silvestris	Protein 1	BAK17630	599	2	3 SLH domains	Cell Surface	This study
Solibacillus silvestris	Protein 2	BAK17631	258	1	None	Multiple Localization Sites (secreted/cell wall)	This study
Staphylococcus aureus	IsdA	ADC37297	350	1	None	Cell Wall (LPXTG anchored)	[16, 37, 67, 69]

Table S1. Putative NEAT proteins

Genus and *species*	Protein Name	Accession #	Amino Acid Length	# NEAT Domains	Other Features	Predicted Localization	Reference
Staphylococcus aureus	IsdB	ADC37296	645	2	None	Cell Wall (LPXTG anchored)	[16, 21, 37, 43]
Staphylococcus aureus	IsdC	ADC37298	227	1	None	Cell Wall (LPXTG anchored)	[16, 31, 58, 69]
Staphylococcus aureus	IsdH	ADC37900	891	3	None	Cell Wall (LPXTG anchored)	[16, 29, 41, 42, 82]
Staphylococcus capitis	Protein 1	EEE48922	509	1	None	Cell Wall (LPXTG anchored)	This study
Staphylococcus capitis	Protein 2	ZP_03613761	201	1	None	Multiple Localization Sites (secreted/cell wall)	This study
Staphylococcus capitis	Protein 3	EEE48956	359	1	None	Multiple Localization Sites (secreted/cell wall)	This study
Staphylococcus caprae	Protein 1	EFS17233	1261	3	None	Cell Wall (LPXTG anchored)	This study
Staphylococcus caprae	Protein 2	EFS17234	504	1	None	Cell Wall (LPXTG anchored)	This study
Staphylococcus caprae	Protein 3	EFS17235	155	1	None	Secreted	This study
Staphylococcus caprae	Protein 4	EFS17236	360	1	None	Multiple Localization Sites (secreted/cell wall)	This study

Table S1. Putative NEAT proteins

Genus and *species*	Protein Name	Accession #	Amino Acid Length	# NEAT Domains	Other Features	Predicted Localization	Reference
Staphylococcus epidermidis	Protein 1	EES40324	718	1	None	Cell Wall (LPXTG anchored)	This study
Staphylococcus epidermidis	Protein 2	EES40325	493	1	None	Cell Wall (LPXTG anchored)	This study
Staphylococcus epidermidis	Protein 3	EES40327	206	1	None	Secreted	This study
Staphylococcus epidermidis	Protein 4	EES40328	355	1	None	Multiple Localization Sites (secreted/cell wall)	This study
Staphylococcus lentus	Protein 1	WP_017000196	481	1	YSIRK-signal peptide	Cell Surface	This study
Staphylococcus lentus	Protein 2	WP_016999526	235	1	None	Multiple Localization Sites (secreted/cell wall)	This study
Staphylococcus lentus	Protein 3	WP_016999525	794	3	None	Cell Surface	This study
Staphylococcus lugdunensis	Protein 1	ADC86209	690	2	None	Cell Wall (LPXTG anchored)	This study
Staphylococcus lugdunensis	Protein 2	ADC86213	646	2	None	Cell Wall (LPXTG anchored)	This study
Staphylococcus lugdunensis	Protein 3	ADC86214	228	1	None	Multiple Localization Sites (secreted/cell wall)	This study

Table S1. Putative NEAT proteins

Genus and *species*	Protein Name	Accession #	Amino Acid Length	# NEAT Domains	Other Features	Predicted Localization	Reference
Staphylococcus lugdunensis	Protein 4	ADC86215	465	1	None	Multiple Localization Sites (secreted/cell wall)	This study
Staphylococcus massiliensis	Protein 1	WP_009382383	199	1	None	Cell Surface	This study
Staphylococcus simulans	Protein 1	WP_002480440	485	2	None	Cell Surface	This study
Staphylococcus simulans	Protein 2	WP_002480439	221	1	None	Multiple Localization Sites (secreted/cell wall)	This study
Staphylococcus simulans	Protein 3	WP_002480438	296	1	None	Cell Wall (LPXTG anchored)	This study
Staphylococcus simulans	Protein 4	WP_002480437	349	1	None	Cell Surface	This study
Streptococcus dysgalactiae	Protein 1	EFY03540	1288	2	LRR 4 (2 copies)	Multiple Localization Sites (secreted/cell wall)	This study
Streptococcus equi	Protein 1	CAX00339	1249	2	LRR 4 (2 copies)	Multiple Localization Sites (secreted/cell wall)	This study
Streptococcus ictaluri	Protein 1	WP_008087116	780	1	Domain of unknown function (DUF1533); 3 copy LRR	Cell Surface	This study
Streptococcus iniae	Protein 1	YP_008057484	1252	2	Domain of unknown function (DUF1533); 2 copy LRR	Cell Surface	This study

Table S1. Putative NEAT proteins

Genus and *species*	Protein Name	Accession #	Amino Acid Length	# NEAT Domains	Other Features	Predicted Localization	Reference
Streptococcus pyogenes	Shr	ABW80932	1275	2	LRR 4 (2 copies)	Membrane anchored	[23, 25]
Streptococcus pyogenes	Shp	ABI79309	291	1	None	Secreted	[23]
Syntrophobotulus glycolicus	Protein 1	ADY55784	4339	6	4 LRR4s (2 copies each); 3 putative cell wall binding repeat 2	Multiple Localization Sites (secreted/cell wall)	This study
Weissella confusa	Protein 1	WP_003609864	258	1	None	Multiple Localization Sites (secreted/cell wall)	This study
Weissella paramesenteroides	Protein 1	EER75028	885	1	None	Multiple Localization Sites (secreted/cell wall)	This study
