# Supplementary material for: Molecular and Evolutionary Analysis of NEAr-Iron Transporter (NEAT) Domains
Source: PLoS One. 2014 Aug 25;9(8):e104794. doi: 10.1371/journal.pone.0104794 (PMC4143258; doi:10.1371/journal.pone.0104794)
Supplement: Table S2 — List of organisms carrying genes predicted to encode NEAT domain proteins arranged by phylum, class, order, family, genus and species. (PDF) [file pone.0104794.s003.pdf]

Table S2. Taxonomic classification of bacterial species identified in this study to harbor putative NEAT domains.

Phylum	Class	Order	Family	*Genus Species*	# NEAT Proteins	# NEAT Domains
Actinobacteria	Actinobacteria	Coriobacteriales	Coriobacteriaceae	*Eggerthella sp. CAG:298*	1	2
Firmicutes	Bacilli	Bacillales	Bacillaceae	*Bacillus anthracis*	6	10
Firmicutes	Bacilli	Bacillales	Bacillaceae	*Bacillus cereus*	4	8
Firmicutes	Bacilli	Bacillales	Bacillaceae	*Bacillus clausii*	2	5
Firmicutes	Bacilli	Bacillales	Bacillaceae	*Bacillus halodurans*	2	6
Firmicutes	Bacilli	Bacillales	Bacillaceae	*Bacillus mycoides*	5	10
Firmicutes	Bacilli	Bacillales	Bacillaceae	*Bacillus pumilus*	1	3
Firmicutes	Bacilli	Bacillales	Bacillaceae	*Bacillus sp. GeD10*	5	9
Firmicutes	Bacilli	Bacillales	Bacillaceae	*Bacillus thuringiensis*	5	9
Firmicutes	Bacilli	Bacillales	Bacillaceae	*Bacillus weihenstephanensis*	5	10
Firmicutes	Bacilli	Bacillales	Bacillaceae	*Lysinibacillus sphaericus*	4	5
Firmicutes	Bacilli	Bacillales	Listeriaceae	*Listeria innocua*	2	4
Firmicutes	Bacilli	Bacillales	Listeriaceae	*Listeria ivanovii*	2	3
Firmicutes	Bacilli	Bacillales	Listeriaceae	*Listeria marthii*	1	1
Firmicutes	Bacilli	Bacillales	Listeriaceae	*Listeria monocytogenes*	5	8
Firmicutes	Bacilli	Bacillales	Listeriaceae	*Listeria seeligeri*	3	6
Firmicutes	Bacilli	Bacillales	Listeriaceae	*Listeria welshimeri*	2	4
Firmicutes	Bacilli	Bacillales	Paenibacillaceae	*Cohnella laeviribosi*	3	5
Firmicutes	Bacilli	Bacillales	Paenibacillaceae	*Paenibacillus daejeonensis*	1	1

Table S2. Taxonomic classification of bacterial species identified in this study to harbor putative NEAT domains.

Phylum	Class	Order	Family	*Genus Species*	# NEAT Proteins	# NEAT Domains
Firmicutes	Bacilli	Bacillales	Paenibacillaceae	*Paenibacillus larvae*	2	3
Firmicutes	Bacilli	Bacillales	Paenibacillaceae	*Paenibacillus massiliensis*	3	9
Firmicutes	Bacilli	Bacillales	Paenibacillaceae	*Paenibacillus peoriae*	4	10
Firmicutes	Bacilli	Bacillales	Paenibacillaceae	*Paenibacillus polymyxa*	5	20
Firmicutes	Bacilli	Bacillales	Paenibacillaceae	*Paenibacillus sp. Aloe-11*	5	8
Firmicutes	Bacilli	Bacillales	Paenibacillaceae	*Paenibacillus sp. JDR-2*	4	5
Firmicutes	Bacilli	Bacillales	Planococcaceae	*Solibacillus silvestris*	2	3
Firmicutes	Bacilli	Bacillales	Staphylococcaceae	*Staphylococcus aureus*	4	7
Firmicutes	Bacilli	Bacillales	Staphylococcaceae	*Staphylococcus capitis*	3	3
Firmicutes	Bacilli	Bacillales	Staphylococcaceae	*Staphylococcus caprae*	4	6
Firmicutes	Bacilli	Bacillales	Staphylococcaceae	*Staphylococcus epidermidis*	4	4
Firmicutes	Bacilli	Bacillales	Staphylococcaceae	*Staphylococcus lentus*	3	5
Firmicutes	Bacilli	Bacillales	Staphylococcaceae	*Staphylococcus lugdunensis*	4	6
Firmicutes	Bacilli	Bacillales	Staphylococcaceae	*Staphylococcus massiliensis*	1	1
Firmicutes	Bacilli	Bacillales	Staphylococcaceae	*Staphylococcus simulans*	4	5
Firmicutes	Bacilli	Lactobacillales	Carnobacteriaceae	*Carnobacterium maltaromaticum*	3	9
Firmicutes	Bacilli	Lactobacillales	Lactobacillaceae	*Lactobacillus acidipiscis*	1	1
Firmicutes	Bacilli	Lactobacillales	Lactobacillaceae	*Lactobacillus brevis*	3	3
Firmicutes	Bacilli	Lactobacillales	Lactobacillaceae	*Lactobacillus buchneri*	1	1

Table S2. Taxonomic classification of bacterial species identified in this study to harbor putative NEAT domains.

Phylum	Class	Order	Family	*Genus Species*	# NEAT Proteins	# NEAT Domains
Firmicutes	Bacilli	Lactobacillales	Lactobacillaceae	*Lactobacillus coryniformis*	3	8
Firmicutes	Bacilli	Lactobacillales	Lactobacillaceae	*Lactobacillus crispatus*	1	1
Firmicutes	Bacilli	Lactobacillales	Lactobacillaceae	*Lactobacillus equicursoris*	1	1
Firmicutes	Bacilli	Lactobacillales	Lactobacillaceae	*Lactobacillus fermentum*	2	2
Firmicutes	Bacilli	Lactobacillales	Lactobacillaceae	*Lactobacillus gastricus*	1	1
Firmicutes	Bacilli	Lactobacillales	Lactobacillaceae	*Lactobacillus gigeriorum*	1	1
Firmicutes	Bacilli	Lactobacillales	Lactobacillaceae	*Lactobacillus hilgardii*	1	1
Firmicutes	Bacilli	Lactobacillales	Lactobacillaceae	*Lactobacillus mali*	1	1
Firmicutes	Bacilli	Lactobacillales	Lactobacillaceae	*Lactobacillus otakiensis*	1	1
Firmicutes	Bacilli	Lactobacillales	Lactobacillaceae	*Lactobacillus parabrevis*	1	1
Firmicutes	Bacilli	Lactobacillales	Lactobacillaceae	*Lactobacillus parafarraginis*	1	1
Firmicutes	Bacilli	Lactobacillales	Lactobacillaceae	*Lactobacillus pasteurii*	1	1
Firmicutes	Bacilli	Lactobacillales	Leuconostocaceae	*Leuconostoc argentinum*	1	1
Firmicutes	Bacilli	Lactobacillales	Leuconostocaceae	*Leuconostoc citreum*	1	1
Firmicutes	Bacilli	Lactobacillales	Leuconostocaceae	*Leuconostoc kimchii*	1	1
Firmicutes	Bacilli	Lactobacillales	Leuconostocaceae	*Leuconostoc pseudomesenteroides*	1	1
Firmicutes	Bacilli	Lactobacillales	Leuconostocaceae	*Weissella confusa*	1	1
Firmicutes	Bacilli	Lactobacillales	Leuconostocaceae	*Weissella paramesenteroides*	1	1
Firmicutes	Bacilli	Lactobacillales	Streptococcaceae	*Streptococcus dysgalactiae*	1	2

Table S2. Taxonomic classification of bacterial species identified in this study to harbor putative NEAT domains.

Phylum	Class	Order	Family	*Genus Species*	# NEAT Proteins	# NEAT Domains
Firmicutes	Bacilli	Lactobacillales	Streptococcaceae	*Streptococcus equi*	1	2
Firmicutes	Bacilli	Lactobacillales	Streptococcaceae	*Streptococcus ictaluri*	1	1
Firmicutes	Bacilli	Lactobacillales	Streptococcaceae	*Streptococcus iniae*	1	2
Firmicutes	Bacilli	Lactobacillales	Streptococcaceae	*Streptococcus pyogenes*	2	3
Firmicutes	Clostridia	Clostridiales	Clostridiaceae	*Clostridium bartlettii*	2	4
Firmicutes	Clostridia	Clostridiales	Clostridiaceae	*Clostridium botulinum*	5	12
Firmicutes	Clostridia	Clostridiales	Clostridiaceae	*Clostridium butyricum*	1	7
Firmicutes	Clostridia	Clostridiales	Clostridiaceae	*Clostridium celatum*	2	3
Firmicutes	Clostridia	Clostridiales	Clostridiaceae	*Clostridium chauvoei*	2	4
Firmicutes	Clostridia	Clostridiales	Clostridiaceae	*Clostridium methylpentosum*	1	1
Firmicutes	Clostridia	Clostridiales	Clostridiaceae	*Clostridium nexile*	1	1
Firmicutes	Clostridia	Clostridiales	Clostridiaceae	*Clostridium novyi*	4	8
Firmicutes	Clostridia	Clostridiales	Clostridiaceae	*Clostridium perfringens*	2	5
Firmicutes	Clostridia	Clostridiales	Clostridiaceae	*Clostridium ramosum*	2	4
Firmicutes	Clostridia	Clostridiales	Clostridiaceae	*Clostridium spiroforme*	1	3
Firmicutes	Clostridia	Clostridiales	Clostridiaceae	*Clostridium sporogenes*	2	8
Firmicutes	Clostridia	Clostridiales	Clostridiaceae	*Clostridium tetani*	2	8
Firmicutes	Clostridia	Clostridiales	Lachnospiraceae	*bacterium*	1	1
Firmicutes	Clostridia	Clostridiales	Lachnospiraceae	*Ruminococcus gnavus*	1	1

Table S2. Taxonomic classification of bacterial species identified in this study to harbor putative NEAT domains.

Phylum	Class	Order	Family	*Genus Species*	# NEAT Proteins	# NEAT Domains
Firmicutes	Clostridia	Clostridiales	Lachnospiraceae	*Anaerostipes hadrus*	3	3
Firmicutes	Clostridia	Clostridiales	Peptococcaceae	*Syntrophobotulus glycolicus*	1	6
Firmicutes	Clostridia	Clostridiales	Peptostreptococcaceae	*Eubacterium yurii*	1	1
Firmicutes	Clostridia	Clostridiales	Peptostreptococcaceae	*Peptostreptococcus anaerobius*	1	1
Firmicutes	Erysipelotrichia	Erysipelotrichales	Erysipelotrichaceae	*Coprobacillus sp. D7*	2	4
Tenericutes	Mollicutes	Unclassified	Unclassified	*Mollicutes bacterium D7*	2	4
					185	343
